# LIMA1 O‐GlcNAcylation Promotes Hepatic Lipid Deposition through Inducing β‐catenin‐Regulated FASn Expression in Metabolic Dysfunction‐Associated Steatotic Liver Disease

**DOI:** 10.1002/advs.202415941

**Published:** 2025-02-08

**Authors:** Fuji Yang, Yifei Chen, Guojun Zheng, Kefeng Gu, Lin Fan, Tingfen Li, Ling Zhu, Yongmin Yan

**Affiliations:** ^1^ Department of Laboratory Medicine Wujin Hospital Affiliated with Jiangsu University Jiangsu University Changzhou 213017 China; ^2^ Department of Laboratory Medicine School of Medicine Jiangsu University Zhenjiang 212013 China; ^3^ Department of Laboratory Medicine The Third People's Hospital of Changzhou Changzhou 213017 China; ^4^ Changzhou Key Laboratory of Exosome Foundation and Transformation Application Wujin Hospital Affiliated with Jiangsu University Jiangsu University Changzhou 213017 China; ^5^ Wujin Institute of Molecular Diagnostics and Precision Cancer Medicine of Jiangsu University Jiangsu University Changzhou 213017 China; ^6^ Department of laboratory medicine The Second People's Hospital of Changzhou Changzhou 213614 China

**Keywords:** LIMA1, lipid deposition, MASLD, O‐GlcNAcylation, small extracellular vesicles

## Abstract

Hepatic lipid deposition is a key factor in progressing metabolic dysfunction‐associated steatotic liver disease (MASLD). This study investigates the impact of the LIM domain and actin‐binding protein 1 (LIMA1) on hepatic steatotic in MASLD and explore the underlying mechanisms. Increased levels of LIMA1 is observed in both serum and serum sEV of metabolic dysfunction‐associated steatohepatitis (MASH) patients compared to healthy controls, with AUROC values of 0.76 and 0.86, respectively. Furthermore, increased LIMA1 O‐GlcNAcylation is observed in mouse models of MASLD, and steatotic hepatocytes. Mechanistic studies revealed that steatosis upregulated Host cell factor 1 (HCF1) and O‐GlcNAc transferase (OGT) expression, leading to catalyzed O‐GlcNAcylation at the T662 site of LIMA1 and subsequent inhibition of its ubiquitin‐dependent degradation. O‐GlcNAcylation of LIMA1 enhances hepatocyte lipid deposition by activating β‐catenin/FASn‐associated signaling. Additionally, compared with their AAV8‐TBG‐LIMA1‐WT counterparts, AAV8‐TBG‐LIMA1^ΔT662^ injection exhibited decreases in systemic insulin resistance, steatosis severity, inflammation and fibrosis in HFD‐fed and CDAHFD‐fed LIMA1 HKO (hepatocyte‐specific knockout) mice. Moreover, LTH‐sEV‐mediated delivery of LIMA1 promoted MASLD progression by promoting hepatic stellate cell (HSC) activation. The findings suggest that serum sEV LIMA1 may be a potential noninvasive biomarker and therapeutic target for individuals with MASH.

## Graphical abstract

Early diagnosis and treatment of metabolic dysfunction‐associated steatotic liver disease (MASLD) remains a significant challenge. This study indicates that serum sEV LIMA1 may serve as a noninvasive biomarker for MASH. The HCF1/OGT complex induces O‐GlcNAcylation of the LIMA1 protein, and subsequently promotes lipid accumulation in hepatocytes by activating the β‐catenin/FASn signaling pathway. Inhibiting LIMA1 O‐GlcNAcylation protects mice against diet‐induced MASLD.

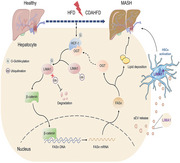

## 1 Introduction

Metabolic dysfunction‐associated steatotic liver disease (MASLD) is a chronic liver disease affecting ≈30% of adults worldwide.^[^ ^1^ ^]^ Metabolic dysfunction‐associated steatohepatitis (MASH) represents the active phase of MASLD, characterized by inflammation and faster fibrosis progression in the liver.^[^ ^2^ ^]^ Currently, there are no optimal diagnostic methods for MASH, except for the highly invasive liver biopsy.^[^ ^3^ ^]^ Additionally, the FDA has only approved resmetirom for the prevention or treatment of MASH.^[^ ^4^, ^5^ ^]^ Therefore, it is crucial to understand the underlying mechanism of MASLD progression and develop specific non‐invasive biomarkers and new treatment strategies for MASLD.

Small extracellular vesicles (sEV) are membranous vesicles originating from different cells in the liver.^[^ ^6^ ^]^ Central to the production of these sEV are lipotoxic hepatocytes (LTH‐sEV), and the sEV released by these cells provide a tangible link between the initial lipid accumulation in MASLD and the subsequent development of hepatic inflammation and fibrosis.^[^ ^7^ ^]^ As serum levels of LTH‐sEV increase with the progression of MASH, LTH‐sEV show great potential as noninvasive diagnostic biomarkers for MASH.^[^ ^8^, ^9^ ^]^ The proteomic signatures in circulating sEV that allowed differentiation between MASH and healthy controls.^[^ ^8^ ^]^ Identification of specific metabolism‐associated proteins in LTH‐sEV may improve the diagnosis and treatment of MASH.

O‐GlcNAcylation is a significant post‐translational modification of proteins that can impact protein stability and activity.^[^ ^10^ ^]^ It plays a vital role in liver diseases, controlling the expression of hepatic fatty acid synthase and promoting the progression of hepatic steatosis and metabolic dysfunction‐associated steatotic liver (MASL) to MASH, as well as the progression of hepatitis B virus‐related hepatocellular carcinoma (HCC).^[^ ^11^, ^12^ ^]^ Abnormal O‐GlcNAcylation also contributes to insulin resistance, lipid deposition, inflammatory damage, fibrosis, and tumorigenesis in liver tissue.^[^ ^13^, ^14^ ^]^ Elucidating the essential proteins that regulate hepatocyte steatosis and their O‐GlcNAcylation modification mechanisms will help promote the development of new and more effective treatments for MASLD.

LIM domain and actin‐binding protein 1 (LIMA1), also known as epithelial protein lost in tumors (EPLIN), plays a critical role in regulating the actin cytoskeleton.^[^ ^15^ ^]^ Recent research has highlighted LIMA1's involvement in key cellular processes such as cell migration, cytoskeletal dynamics, cell cycle regulation, gene regulation, and angiogenesis.^[^ ^15^, ^16^ ^]^ While past studies have shown LIMA1's impact on cholesterol uptake and cellular metabolism,^[^ ^16^, ^17^ ^]^ its influence on regulating hepatocyte steatosis and MASLD progression requires further investigation.

This study explores the potential of serum sEV LIMA1 as a noninvasive biomarker for diagnosing MASH and delves into the role of LIMA1 in lipid accumulation in steatotic hepatocytes and MASLD progression. The research reveals that O‐GlcNAc transferase (OGT)/Host cell factor 1 (HCF1)‐induced O‐GlcNAcylation stabilizes LIMA1 by reducing its ubiquitination, shedding light on the impact of LIMA1 O‐GlcNAcylation in regulating β‐catenin/FASn expression to enhance lipid accumulation. FASn further enhances protein O‐GlcNAcylation in hepatocytes by up‐regulating OGT, thereby establishing a positive feedback loop that enables LIMA1 to increase O‐GlcNAcylation levels within hepatocytes. Additionally, the study reveals that lipotoxic hepatocytes‐derived small extracellular vesicles (LTH‐sEV)‐mediated delivery of LIMA1 enhanced MASLD progression by promoting hepatic stellate cell (HSC) activation. These findings suggest that LIMA1 could serve as a valuable biomarker and therapeutic target for MASH.

## 2 Results

### 2.1 Serum sEV LIMA1 Discriminated MASH from Healthy Control

**Figure 1:**
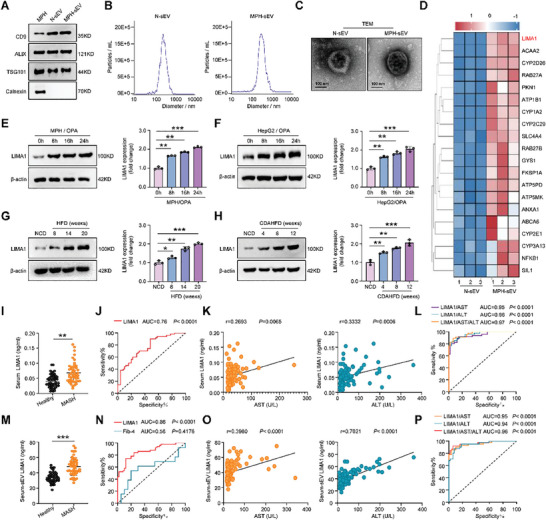
LC‐MS/MS analysis of N‐sEV and MPH‐sEV and predictive value of serum‐derived small extracellular vesicles (sEV) LIMA1 for MASH patients. A) Western blot analysis of CD9, ALIX, TSG101, and Calnexin in mouse primary hepatocytes (MPH), normal MPH‐derived from small extracellular vesicles (N‐sEV), and steatotic MPH‐derived from sEV (MPH‐sEV). B) Representative result of nanoparticle tracking analyses (NTA) of N‐sEV and MPH‐sEV. C) Representative transmission electron microscope (TEM) image of N‐sEV and MPH‐sEV. Scale bar, 100 nm. D) Proteomic profiling of MPH‐sEV as compared to N‐sEV was analyzed by LC‐MS/MS (*n* = 3). Heat map of the top 20 differentially expressed proteins in the metabolism signaling pathway. E, F) Western blot analysis of LIMA1 in MPH and HepG2 cells treated with oleic acid and palmitic acid (OPA) for 0, 8, 16, and 24 h (*n* = 3). G, H) Western blot analysis of LIMA1 in livers from normal chow diet (NCD)‐fed mice, HFD‐fed mice, and CDAHFD‐fed mice at different weeks (*n* = 3). I) Relative serum LIMA1 levels in patients with MASH and healthy control (*n* = 50). J) Receiver operating characteristic curve of the performance of serum LIMA1 levels in diagnosing MASH among 50 patients with biopsy‐proven MASLD. The AUROC is shown. K) Correlation of serum LIMA1 levels with serum aspartate transaminase (AST) and alanine aminotransferase (ALT) levels. L) Receiver operating characteristic curve of the performance of serum LIMA1 levels combined with other clinical risk factors in diagnosing MASH among 50 patients with biopsy‐proven MASLD. The AUROC is shown. M) Relative serum‐derived sEV LIMA1 levels in patients with MASH and healthy control (*n* = 50). N) Receiver operating characteristic curve of the performance of serum‐derived sEV LIMA1 levels and Fib‐4 (Fib‐4 = age×AST/PLT×√ALT) in diagnosing MASH among 50 patients with biopsy‐proven MASLD. The AUROC is shown. O) Correlation of serum‐derived sEV LIMA1 levels with serum AST and ALT levels. P) Receiver operating characteristic curve of the performance of serum‐derived sEV LIMA1 levels combined with other clinical risk factors in diagnosing MASH among 50 patients with biopsy‐proven MASLD. The data were plotted as Mean ± SEM. * *p *< 0.05, ** *p* < 0.01, *** *p* < 0.001 by Student's *t*‐test for I and M; by one‐way ANOVA for E, F, G and H.

We initially investigated potential active molecules in lipotoxic mouse primary hepatocytes‐derived small extracellular vesicles (MPH‐sEV) associated with metabolic dysfunction‐associated steatohepatitis (MASH) progression. Both normal mouse primary hepatocytes‐derived sEV (N‐sEV) and lipotoxic MPH‐sEV exhibited the presence of common extracellular vesicle markers such as CD9, ALIX, and TSG101, with an average particle size of 110 nm. Notably, they did not express the endoplasmic reticulum protein Calnexin (**Figure** **1**A–C). Mass spectrometry analysis revealed a significant increase in more than 20 proteins, including LIMA1, in MPH‐sEV compared to N‐sEV (Figure 1D). Moreover, the expression of LIMA1 protein was notably elevated in OPA‐damaged MPH and HepG2 cells (Figure 1E,F), as well as in fat vacuolated hepatocytes and activating hepatic stellate cells (HSC) within the liver tissue of HFD‐fed and CDAHFD‐fed mice (Figure 1G,H; Figure S1A–F, Supporting Information).

We then compared the levels of LIMA1 in the serum and serum sEV of 50 MASH patients and 50 healthy individuals. Serum‐derived sEV from MASH patients and healthy controls expressed CD9, ALIX, and TSG101, with an average particle size of 110 nm (Figure S1G–I, Supporting Information). The results revealed that the LIMA1 level in the serum of MASH patients was significantly higher compared to healthy controls (Figure 1I). The AUROC analysis demonstrated that serum LIMA1 had a diagnostic efficiency of 0.76 for MASH (Figure 1J). Furthermore, serum LIMA1 showed moderate correlations with serum aspartate aminotransferase (AST) (r = 0.2693, *p* = 0.0065) and alanine aminotransferase (ALT) (r = 0.3332, *p* = 0.0006) (Figure 1K). Combining serum LIMA1 with AST, ALT, and AST/ALT in a clinical prediction model significantly increased the AUROC from 0.76 to 0.95‐0.97 (Figure 1L). Similarly, the level of LIMA1 in serum sEV of MASH patients was higher than in healthy controls (Figure 1M), with a diagnostic efficiency of 0.86 (Figure 1N), showing better performance than the current diagnostic indicator Fib‐4 (Figure 1N). LIMA1 in serum‐derived sEV exhibited strong correlations with AST (r = 0.3980, *p* < 0.0001) and ALT (r = 0.7021, *p* < 0.0001) (Figure 1O). Integration of LIMA1 in serum‐derived sEV with AST, ALT, and AST/ALT in various clinical prediction models significantly increased the AUROC from 0.86 to 0.94‐0.96 (Figure 1P). Moreover, serum LIMA1 levels was upregulated in HFD‐fed and CDAHFD‐fed LIMA1 fl/fl mice, but downregulated in LIMA1 HKO mice, indicating that hepatocytes secreted LIMA1 into mouse serum (Figure S1J,K, Supporting Information). A total of 50 healthy individuals and 50 MASH patients were categorized into gender groups, and the results indicated that no significant differences in serum and serum sEV LIMA1 levels between male and female MASH patients (Figure S1L,M, Supporting Information). These results suggest that both serum LIMA1 and serum sEV LIMA1 indicate liver injury in MASH patients, with sEV LIMA1 being a better diagnostic indicator.

### 2.2 Steatosis‐Induced O‐GlcNAcylation at Threonine 662 of LIMA1 and Upregulated LIMA1 Expression in Hepatocytes

**Figure 2:**
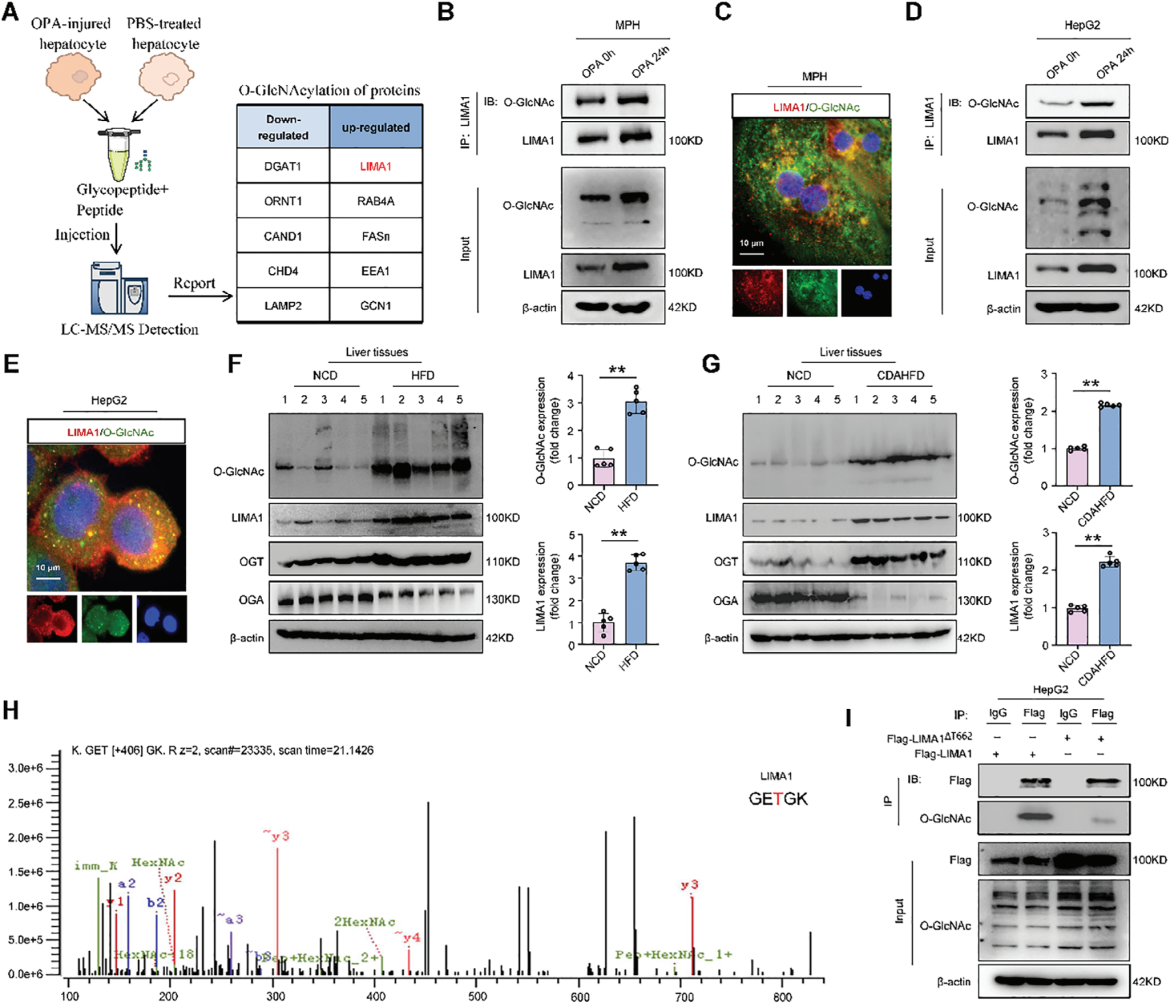
Lipotoxicity induces O‐GlcNAcylation of LIMA1 threonine 662 and upregulates LIMA1 protein levels in hepatocytes. A) Mass spectrometry analysis of proteins undergoing O‐GlcNAcylation in normal human hepatocytes and OPA‐treated human hepatocytes for 24 h. B) Interaction between LIMA1 and O‐GlcNAc by Co‐IP in OPA‐treated MPH at 0 and 24 h. C) Immunofluorescence showing LIMA1 (red) and O‐GlcNAc (green) colocalization in MPH. Scale bars, 10 µm. D) Interaction between LIMA1 and O‐GlcNAc by Co‐IP in OPA‐treated HepG2 cells at 0 and 24 h. E) Immunofluorescence showing LIMA1 (red) and O‐GlcNAc (green) colocalization in HepG2 cells. Scale bars, 10 µm. F, G) Western blot analysis of LIMA1, O‐GlcNAc, OGT, and OGA in livers from NCD‐fed mice, HFD‐fed mice, and CDAHFD‐fed mice (*n* = 5). H) Mass spectrometry of O‐GlcNAcylated peptides from LIMA1 in OPA‐treated HepG2 cells. I) Interaction between LIMA1 and O‐GlcNAc by Co‐IP in HepG2 cells transfected with Flag‐LIMA1 and Flag‐LIMA1^ΔT662^. After immunoprecipitation with Flag antibodies, immunoprecipitates were analyzed using an anti‐O‐GlcNAc antibody. The data were plotted as Mean ± SEM. ** *p* < 0.01 by Student's *t*‐test.

O‐GlcNAcylation is a post‐translational modification of proteins that enhances protein stability and plays a crucial role in regulating steatosis and the progression of MASLD.^[^ ^14^, ^18^ ^]^ We investigated the levels of protein O‐GlcNAcylation in OPA‐injured human hepatocytes using a lectin chip. Our results showed a significant increase in O‐GlcNAcylation of LIMA1 in steatotic human hepatocytes compared to normal human hepatocytes (**Figure** **2**A). Further experiments, including co‐immunoprecipitation and fluorescence co‐localization, revealed that OPA treatment increased protein expression levels of O‐GlcNAc and LIMA1 in both MPH and HepG2 cells (Figure 2B–E). There was also a positive correlation between LIMA1 expression and O‐GlcNAcylation (Figure 2B–E). Moreover, higher levels of O‐GlcNAc, LIMA1, OGT proteins and lower levels of OGA protein were observed in liver tissues of HFD‐fed and CDAHFD‐fed mice (Figure 2F,G; Figure S1N,O, Supporting Information). Mass spectrometry analysis identified threonine 662 (T662) as the site of O‐GlcNAcylation on LIMA1 (Figure 2H). To confirm the importance of T662 in LIMA1 glycosylation, a LIMA1 vector with a T662A mutation (Flag‐LIMA1^ΔT662^) was generated, and co‐immunoprecipitation experiments in HepG2 cells showed a significant reduction in O‐GlcNAcylation levels of LIMA1 in cells expressing Flag‐LIMA1^ΔT662^ compared to Flag‐LIMA1, indicating that T662 is the primary O‐GlcNAcylation site on LIMA1 (Figure 2I). These results suggest that steatosis induces O‐GlcNAcylation at the T662 site of LIMA1, leading to increased LIMA1 protein levels in hepatocytes.

### 2.3 HCF1 and OGT Cooperatively Regulated LIMA1 O‐GlcNAcylation in Steatotic Hepatocytes

**Figure 3:**
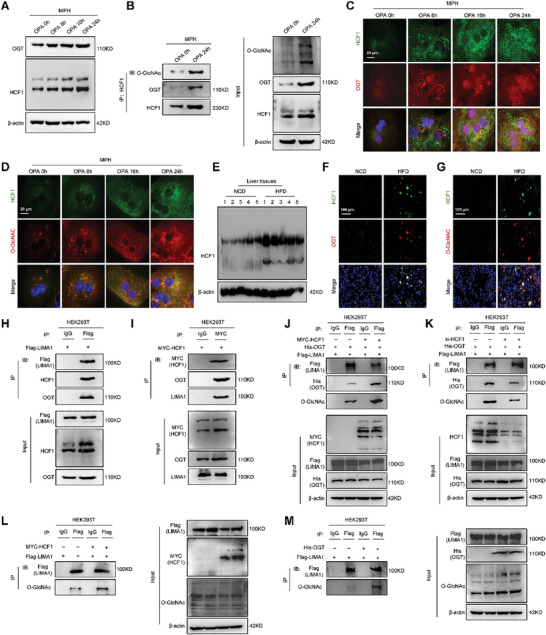
HCF1 and OGT cooperatively promote LIMA1 O‐GlcNAcylation in steatotic hepatocytes. A) Western blot analysis of OGT and HCF1 in OPA‐treated MPH at 0, 8, 16, and 24 h. B) Interaction between O‐GlcNAc, OGT, and HCF1 by Co‐IP in OPA‐treated MPH at 0 and 24 h. C, D) Immunofluorescence showing HCF1 (green), OGT (red) or O‐GlcNAc (red) colocalization in OPA‐treated MPH at 0, 8, 16, and 24 h. Scale bars, 20 µm. E) Western blot analysis of OGT and HCF1 in livers from NCD‐fed mice and HFD‐fed mice at 20 weeks. F, G) Immunofluorescence showing HCF1 (green), OGT (red) or O‐GlcNAc (red) colocalization in livers from NCD‐fed mice and HFD‐fed mice. Scale bars, 100 µm. H) Interaction between LIMA1, HCF1, and OGT by Co‐IP in HEK293T cells transfected with Flag‐LIMA1. I) Interaction between HCF1, OGT, and LIMA1 by Co‐IP in HEK293T cells transfected with MYC‐HCF1. J, K) Co‐IP showing interaction between LIMA1 and OGT or O‐GlcNAc was increased by HCF1 overexpression or decreased by HCF1 knockdown in HEK293T cells co‐transfected with His‐OGT/Flag‐LIMA1 and MYC‐HCF1 or siHCF1, respectively. L, M) Co‐IP showing the interaction between LIMA1 and O‐GlcNAc was increased by HCF1 or OGT overexpression in HEK293T cells co‐transfected with MYC‐HCF1/Flag‐LIMA1 or His‐OGT/Flag‐LIMA1, respectively.

OGT is the primary enzyme responsible for the O‐GlcNAcylation of proteins.^[^ ^19^ ^]^ The transcriptional coregulator HCF1 recruits OGT through O‐GlcNAcylation, leading to the formation of the HCF1/OGT complex, which in turn promotes the O‐GlcNAcylation of target proteins.^[^ ^20^, ^21^ ^]^ It is hypothesized that an increased HCF1/OGT complex may interact with LIMA1 and enhance the O‐GlcNAcylation of LIMA1 in steatotic hepatocytes. Experimental results showed a significant increase in the protein expression levels of HCF1 and OGT in OPA‐treated MPH over time (**Figure** **3**A; Figure S2A,B, Supporting Information). Additionally, it was observed that HCF1 binds to OGT and undergoes O‐GlcNAcylation (Figure 3B–D). Similarly, the expression of HCF1 and OGT was elevated in the liver tissues of HFD‐fed and CDAHFD‐fed mice, with HCF1 undergoing O‐GlcNAcylation (Figure 3E–G; Figure S2C–G, Supporting Information). Further investigations focused on the role of the HCF1/OGT complex in the O‐GlcNAcylation of LIMA1. Co‐IP experiments demonstrated the binding of HCF1 and OGT to Flag‐LIMA1 in HEK293T cells overexpressing Flag‐LIMA1 (Figure 3H). Similarly, LIMA1 and OGT were found to bind to MYC‐HCF1 in HEK293T cells overexpressing MYC‐HCF1 (Figure 3I). In HEK293T cells, overexpression of MYC‐HCF1 facilitated the interaction between Flag‐LIMA1 and His‐OGT or O‐GlcNAc, while HCF1 knockdown reduced this interaction (Figure 3J,K). Moreover, in HEK293T cells transfected with Flag‐LIMA1, the overexpression of MYC‐HCF1 or His‐OGT led to a significant increase in the O‐GlcNAcylation of LIMA1 (Figure 3L,M). These results suggest that HCF1 may enhance the O‐GlcNAcylation of LIMA1 in steatotic hepatocytes by recruiting OGT to form a complex.

### 2.4 O‐GlcNAcylation at T662 Stabilized LIMA1 Protein and Reduced Its Ubiquitination and Degradation

**Figure 4:**
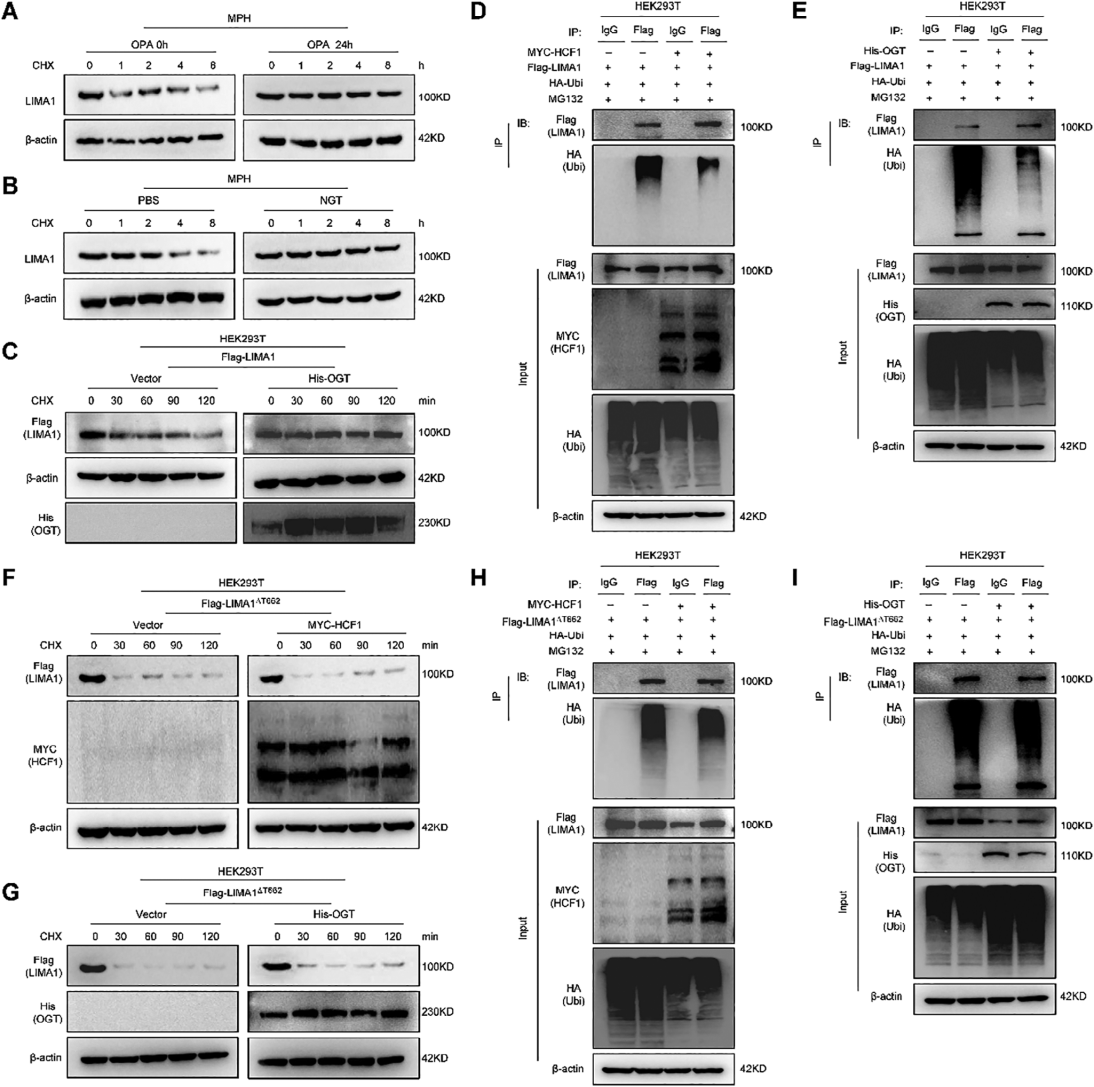
HCF1/OGT complex‐mediated O‐GlcNAcylation of LIMA1 Thr662 enhances LIMA1 protein stability by counteracting its ubiquitylation. A, B) Increased LIMA1 stability was determined by cycloheximide chase (CHX) assay. Following 24 h of PBS/OPA or PBS/NAG‐thiazoline (NGT, 0.008 м) treatment, MPH were exposed to CHX (20 µм) for the indicated times. The cells were then harvested and lysed for western blot analysis. C) Cycloheximide chase assay showing that OGT overexpression increases LIMA1 stabilization. HEK293T cells were transfected with Flag‐LIMA1/His‐OGT and then exposed to CHX (50 µм) for the indicated times. The cells were then harvested and lysed for western blot analysis. D) Co‐IP showing the interaction between LIMA1 and ubiquitin was decreased by HCF1 overexpression in HEK293T cells. The cells were co‐transfected with Flag‐LIMA1/HA‐Ubi/MYC‐HCF1 and treated with MG132 for 4 h. After immunoprecipitation with Flag antibodies, immunoprecipitates were analyzed using an anti‐HA antibody. E) Co‐IP showing the interaction between LIMA1 and ubiquitin was decreased by OGT overexpression in HEK293T cells. The cells were co‐transfected with Flag‐LIMA1/HA‐Ubi/His‐OGT and treated with MG132 for 4 h. F, G) Cycloheximide chase assay shows that HCF1 or OGT overexpression does not change LIMA1 stabilization. HEK293T cells were transfected with Flag‐LIMA1^∆T662^/MCF‐HCF1 or Flag‐LIMA1^∆T662^/His‐OGT, then exposed to CHX (50 µм) for the indicated times. The cells were then harvested and lysed for western blot analysis. H) Co‐IP showing the interaction between LIMA1 and ubiquitin was not changed by HCF1 overexpression in HEK293T cells. The cells were co‐transfected with Flag‐LIMA1^∆T662^/HA‐Ubi/MYC‐HCF1 and treated with MG132 for 4 h. I) Co‐IP showing the interaction between LIMA1 and ubiquitin was not changed by OGT overexpression in HEK293T cells. The cells were co‐transfected with Flag‐LIMA1^∆T662^/HA‐Ubi/His‐OGT and treated with MG132 for 4 h.

We next investigated the impact of O‐GlcNAcylation on the stability of LIMA1 protein in hepatocytes. We observed a significant degradation of LIMA1 protein in MPH treated with PBS and CHX, while treatment with OPA and CHX prevented this degradation by increasing the protein's stability (**Figure** **4**A; Figure S3A, Supporting Information). Additionally, inhibition of O‐GlcNAcase with NAG‐thiazoline (NGT) enhanced LIMA1 O‐GlcNAcylation and further stabilized the protein (Figure 4B; Figure S3B, Supporting Information). These results imply that O‐GlcNAcylation can impede the degradation of LIMA1 protein in OPA‐induced steatotic MPH cells. Furthermore, our investigation into the role of HCF1/OGT in regulating LIMA1 protein degradation through O‐GlcNAcylation revealed that overexpression of MYC‐HCF1 or His‐OGT prolonged the half‐life of Flag‐LIMA1 protein in HEK293T cells (Figure 4C; Figure S3C,D, Supporting Information). Co‐IP analysis also showed that HCF1/OGT overexpression suppressed the ubiquitination of Flag‐LIMA1 protein, enhancing its stability (Figure 4D,E). However, the degradation and ubiquitination of Flag‐LIMA1^ΔT662^ protein were not significantly impacted by MYC‐HCF1 or His‐OGT overexpression (Figure 4F–I; Figure S3E,F, Supporting Information). These findings suggest that HCF1/OGT can regulate O‐GlcNAcylation at the T662 site of LIMA1 protein to prevent its ubiquitination degradation.

### 2.5 LIMA1 O‐GlcNAcylation Increased Lipid Deposition in Hepatocytes by Upregulating FASn Expression

We then investigated the impact of O‐GlcNAcylation on LIMA1 expression and lipid deposition in hepatocytes. Our results indicated that O‐GlcNAcylation induced by NGT in MPH and HepG2 cells led to an increase in LIMA1 expression, despite no significant changes in LIMA1 mRNA levels (Figure S4A,B,D,E, Supporting Information). Moreover, Nile Red staining revealed a notable rise in lipid deposition in NGT‐treated MPH and HepG2 cells (Figure S4C,F, Supporting Information). Subsequently, we examined the influence of LIMA1 on O‐GlcNAcylation‐mediated lipid deposition. Treatment with NGT resulted in increased lipid deposition in HepG2 cells, along with elevated levels of O‐GlcNAc and LIMA1 compared to normal HepG2 cells (Figure S4G,H, Supporting Information). However, silencing LIMA1 inhibited both LIMA1 expression and lipid deposition in NGT‐treated cells (Figure S4G,H, Supporting Information).

**Figure 5:**
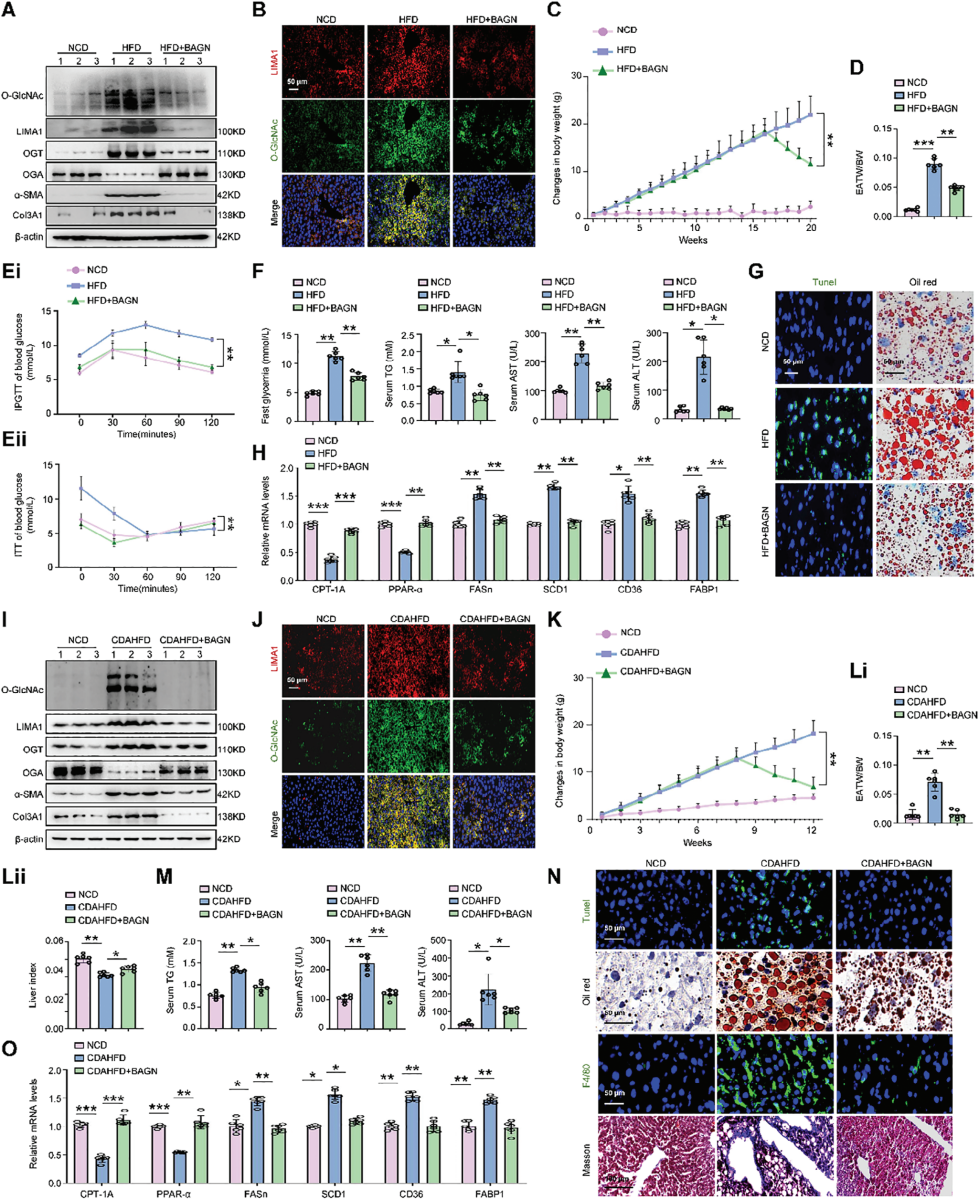
Benzyl‐α‐GalNAc (BAGN) inhibits LIMA1 O‐GlcNAcylation, hepatic steatosis, and liver injury in HFD‐fed mice and CDAHFD‐fed mice. A) Western blot analysis of O‐GlcNAc, LIMA1, OGT, OGA, α‐SMA, and Col3A1 in livers from NCD‐fed mice, HFD‐fed mice, and HFD‐fed mice injected with BAGN. B) Immunofluorescence showing LIMA1 (red) and O‐GlcNAc (green) colocalization in livers from NCD‐fed mice, HFD‐fed mice, and HFD‐fed mice injected with BAGN. Scale bars, 50 µm. C‐F) Changes in body weight, EATW/BW (EATW/BW = relative weight of epididymal adipose tissue to body weight), results of IPGTT and ITT assays, and fast glycemia, serum TG, serum AST, serum ALT levels of NCD‐fed mice, HFD‐fed mice, and HFD‐fed mice injected with BAGN (*n* = 6). G) Tunel (Scale bars, 50 µm) and Oil red O (Scale bars, 50 µm) staining in livers from NCD‐fed mice, HFD‐fed mice, and HFD‐fed mice injected with BAGN. H) Lipometabolic mRNA expression in the liver of NCD‐fed mice, HFD‐fed mice, and HFD‐fed mice injected with BAGN (*n* = 6). I) Western blot analysis of O‐GlcNAc, LIMA1, OGT, OGA, α‐SMA, and Col3A1 in livers from NCD‐fed mice, CDAHFD‐fed mice, and CDAHFD‐fed mice injected with BAGN. J) Immunofluorescence showing LIMA1 (red) and O‐GlcNAc (green) colocalization in livers from NCD‐fed mice, CDAHFD‐fed mice, and CDAHFD‐fed mice injected with BAGN. Scale bars, 50 µm. K‐M) Changes in body weight, liver index, EATW/BW, and serum TG, serum AST, serum ALT levels of NCD‐fed mice, CDAHFD‐fed mice, and CDAHFD‐fed mice injected with BAGN (*n* = 6). N) Tunel (Scale bars, 50 µm), Oil red O (Scale bars, 50 µm), F4/80 (Scale bars, 50 µm), and MASSON (Scale bars, 100 µm) staining in livers from NCD‐fed mice, CDAHFD‐fed mice, and CDAHFD‐fed mice injected with BAGN. O) Lipometabolic mRNA expression in the liver of NCD‐fed mice, CDAHFD‐fed mice, and CDAHFD‐fed mice injected with BAGN (*n* = 6). The data were plotted as Mean ± SEM. * *p *< 0.05, ** *p* < 0.01, *** *p* < 0.001 by one‐way ANOVA.

Additionally, we investigated the impact of the O‐GlcNAcylation inhibitor BAGN on LIMA1 expression and lipid deposition in steatotic hepatocytes, HFD‐fed mice, and CDAHFD‐fed mice. BAGN treatment reduced O‐GlcNAc and LIMA1 expression in OPA‐induced steatotic MPH and HepG2 cells, with no significant changes in LIMA1 mRNA levels (Figure S5A,B,D,E, Supporting Information). Furthermore, BAGN treatment lowered lipid deposition in OPA‐induced steatotic MPH and HepG2 cells (Figure S5C,F, Supporting Information). In HFD‐fed and CDAHFD‐fed mice injected with BAGN, there was a decrease in O‐GlcNAc, LIMA1, OGT, α‐SMA, Col3A1 expression, and increase in OGA expression (**Figure** **5**A,B,I,J; Figure S5G,K, Supporting Information). Decreased body weight and increased liver index were found in HFD‐fed and CDAHFD‐fed mice injected with BAGN, compared to mice fed with HFD or CDAHFD (Figure 5C,D,K,L; Figure S5H, Supporting Information). BAGN administration also showed less weight gain of inguinal adipose tissue in HFD‐fed and CDAHFD‐fed mice (Figure 5D,L). In contrast, food intake was not significantly altered between different groups, and this trend was sustained over the entire treatment period (Figure S5I,L, Supporting Information). Results of IPGTT and ITT showed that BAGN administration was able to bring blood glucose levels back to normal condition quickly in insulin‐and glucose‐injected mice (Figure 5E). Furthermore, elevated liver damage markers such as fast glycemia, serum TG, ALT, and AST in the HFD‐fed and CDAHFD‐fed mice were reversed by BAGN administration (Figure 5F,M). Liver tissues from these mice also showed reduced hepatocyte death and lipid deposition, inflammation, and collagen deposition (Figure 5G,N; Figure S5J, Supporting Information). Analysis of lipometabolic gene expression also validated the impact of BAGN on hepatic steatosis (Figure 5H,O). These findings suggest that increased O‐GlcNAcylation can boost LIMA1 protein expression and facilitate lipid deposition in hepatocytes, with LIMA1 playing a pivotal role in lipid deposition and MASLD progression regulated by O‐GlcNAcylation.

**Figure 6:**
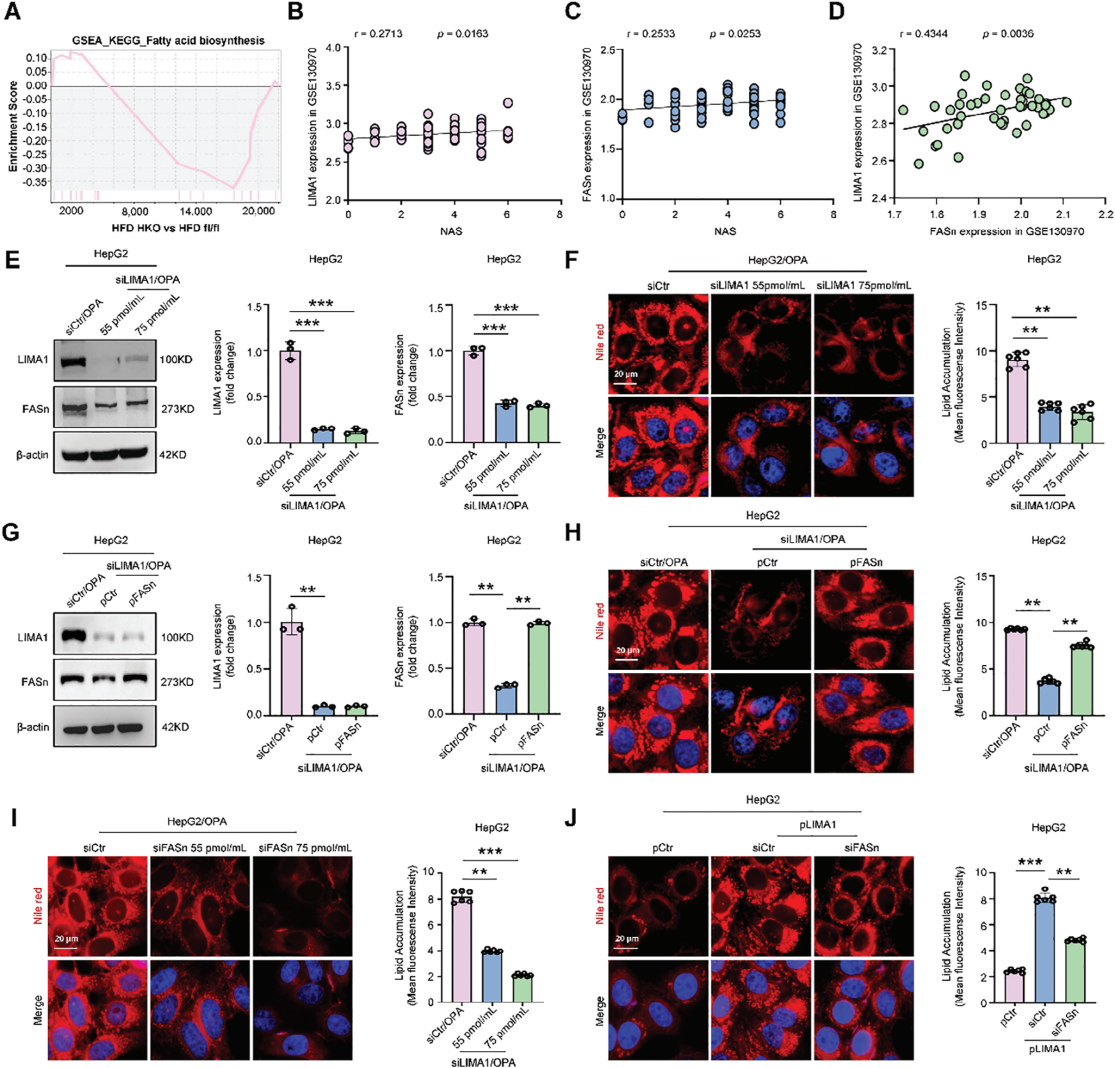
LIMA1 increases lipid deposition in hepatocytes by upregulating FASn expression. A) Gene set enrichment analysis (GSEA) revealed the downregulation of fatty acid biosynthesis‐associated signaling in LIMA1 HKO mice fed with HFD at 20 weeks. B‐D) The gene‐level counts of FASn and LIMA1 from the GEO database (GSE130970) were normalized to log2‐counts per million reads (CPM). Correlation of FASn with liver non‐alcoholic fatty liver activity score (NAS) or LIMA1. E) Western blot analysis of LIMA1 and FASn in OPA‐treated HepG2 cells transfected with siCtr or siLIMA1 (*n* = 3). F) Nile red staining of intracellular lipid droplets in OPA‐treated HepG2 cells transfected with siCtr or siLIMA1 (*n* = 6). Scale bars, 20 µm. G) Western blot analysis of LIMA1 and FASn in siLIMA1 transfected OPA‐treated HepG2 cells co‐transfected with pcDNA3.1 vector (pCtr) or pcDNA3.1‐FASn (pFASn). siCtr transfected OPA‐treated HepG2 cells served as a control (*n* = 3). H) Nile red staining of intracellular lipid droplets in siCtr transfected OPA‐treated HepG2 cells, siLIMA1 transfected OPA‐treated HepG2 cells co‐transfected with pCtr or pFASn (*n* = 6). Scale bars, 20 µm. I) Nile red staining of intracellular lipid deposition in OPA‐treated HepG2 cells transfected with siCtr or FASn siRNA (siFASn) (*n* = 6). Scale bars, 20 µm. J) Nile red staining of intracellular lipid droplets in pcDNA3.1‐LIMA1 (pLIMA1) transfected HepG2 cells co‐transfected with siCtr or siFASn. pCtr transfected HepG2 cells served as a control (*n* = 6). Scale bars, 20 µm. The data were plotted as Mean ± SEM. ** *p* < 0.01, *** *p* < 0.001 by one‐way ANOVA.

Through GSEA analysis of transcript sequencing omics data from HFD‐fed LIMA1 fl/fl and LIMA1 HKO mice, it was discovered that the fatty acid synthesis signaling pathway was downregulated in HFD‐fed LIMA1 HKO mice (**Figure** **6**A). Subsequent comparison of LIMA1 and FASn expression levels in liver samples from healthy controls and MASH patients using the Gene Expression Database (GEO) showed a significant up‐regulation of FASn mRNA expression, which was positively correlated with liver non‐alcoholic fatty liver activity score (NAS) and LIMA1 in the GSE130970 data set (r = 0.2533, *p* = 0.0253 and r = 0.4344, *p* = 0.0036, respectively) (Figure 6B–D). This led to the hypothesis that LIMA1 may play a role in promoting hepatocyte lipid deposition by regulating FASn. To verify this hypothesis, LIMA1 expression plasmid (pLIMA1) and LIMA1 siRNA (siLIMA1) were used to observe the effects of LIMA1 on FASn expression and lipid deposition in hepatocytes. Results demonstrated that LIMA1 knockdown inhibited FASn expression and lipid deposition in OPA‐damaged HepG2 cells (Figure 6E,F). Conversely, FASn overexpression (pFASn) promoted FASn expression and lipid deposition in LIMA1 knockdown steatosis HepG2 cells (Figure 6G,H). Furthermore, Nile red staining revealed that FASn knockdown inhibited lipid deposition in OPA‐damaged HepG2 cells (Figure 6I), while also reversing the hepatocyte lipid deposition induced by LIMA1 overexpression in normal HepG2 cells (Figure 6J). These findings suggest that through the regulation of FASn expression, LIMA1 may enhance lipid deposition in hepatocytes.

### 2.6 LIMA1 Promoted FASn‐Mediated Lipid Deposition by Upregulating β‐catenin in Hepatocytes

**Figure 7:**
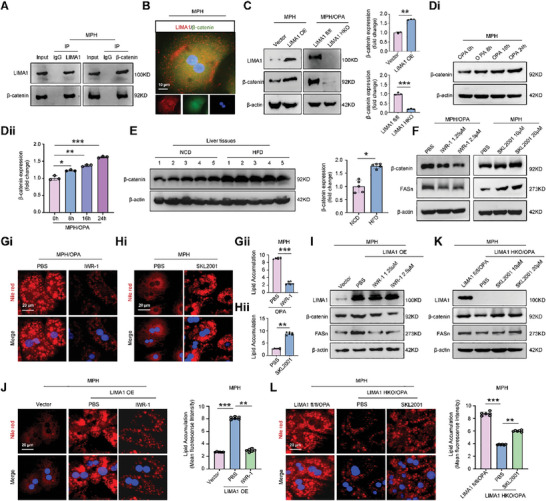
LIMA1 promotes FASn‐mediated lipid deposition by upregulating β‐catenin in hepatocytes. A) Interactions between LIMA1 and β‐catenin by Co‐IP in MPH. B) Immunofluorescence showing LIMA1 (red) and β‐catenin (green) colocalization in MPH. Scale bars, 10 µm. C) Western blot analysis of LIMA1 and β‐catenin in control vector transfected MPH (vector) or pADV‐CMV‐LIMA1‐Flag transfected MPH (LIMA1 OE), and OPA‐treated LIMA1 fl/fl MPH or LIMA1 HKO MPH (*n* = 3). D) Western blot analysis of β‐catenin in OPA‐treated MPH at 0, 8, 16, and 24 h (*n* = 3). E) Western blot analysis of β‐catenin in livers from NCD‐fed mice and HFD‐fed mice at 20 weeks (*n* = 5). F) Western blot analysis of β‐catenin and FASn in PBS or IWR‐1 treated OPA‐injured MPH, and PBS or SKL2001 treated MPH for 24 h, respectively. G, H) Nile red staining of intracellular lipid droplets in PBS or IWR‐1 (2.5 µм) treated OPA‐injured MPH, and PBS or SKL2001 (20 µм) treated MPH for 24 h, respectively (*n* = 6). Scale bars, 20 µm. I) Western blot analysis of LIMA1, β‐catenin, and FASn in LIMA1 OE MPH treated with or without IWR‐1 for 24 h. J) Nile red staining of intracellular lipid droplets in LIMA1 OE MPH treated with or without IWR‐1 (2.5 µм) for 24 h (*n* = 6). Scale bars, 20 µm. K) Western blot analysis of LIMA1, β‐catenin, and FASn in OPA‐treated LIMA1 fl/fl MPH and OPA‐treated LIMA1 HKO MPH treated with or without SKL2001 for 24 h. L) Nile red staining of intracellular lipid deposition in OPA‐treated LIMA1 fl/fl MPH and OPA‐treated LIMA1 HKO MPH treated with or without SKL2001 (20 µм) for 24 h (*n* = 6). Scale bars, 20 µm. The data were plotted as Mean ± SEM. * *p* < 0.05, ** *p* < 0.01, *** *p* < 0.001 by Student's *t*‐test for C, E, G, and H; by one‐way ANOVA for D, J and L.

Since β‐catenin regulated FASn expression plays an important role in regulating lipid deposition and hepatic metabolism,^[^ ^22^, ^23^ ^]^ we are intrigued whether β‐catenin is involved in LIMA1 O‐GlcNAcylation regulated FASn expression. We investigated the potential involvement of β‐catenin in LIMA1 O‐GlcNAcylation‐regulated FASn expression. Results indicated an interaction between LIMA1 and β‐catenin in MPH, with a positive correlation between their expressions (**Figure** **7**A–C). Further analysis revealed an increase in β‐catenin expression in steatotic cells and the livers of HFD‐fed and CDAHFD‐fed mice (Figure 7D,E; Figure S6A, Supporting Information). Inhibition of β‐catenin reduced FASn expression and lipid accumulation, while its activation exacerbated these effects (Figure 7F–H; Figure S6B,C, Supporting Information). These findings suggest that β‐catenin plays a role in promoting FASn expression and lipid deposition in hepatocytes.

The study also examined the regulatory impact of LIMA1 on β‐catenin expression and the influence of β‐catenin activation or inhibition on FASn expression controlled by LIMA1. Results indicated that LIMA1 OE increased levels of β‐catenin and FASn, leading to a significant enhancement of lipid deposition in normal MPH (Figure 7I,J; Figure S6D, Supporting Information). Conversely, inhibiting β‐catenin with IWR‐1 reversed the stimulatory effect of LIMA1 OE on FASn expression and lipid deposition (Figure 7I,J; Figure S6D, Supporting Information). Additionally, LIMA1 HKO decreased β‐catenin and FASn expression, resulting in reduced lipid deposition in OPA‐injured MPH (Figure 7K,L; Figure S6E, Supporting Information). Treatment with SKL2001 counteracted the inhibitory effect of LIMA1 HKO on FASn expression and lipid deposition (Figure 7K, L; Figure S6E, Supporting Information). These findings suggest that LIMA1 may facilitate hepatocyte lipid deposition by activating the β‐catenin/FASn pathway.

### 2.7 Inhibiting LIMA1 O‐GlcNAcylation Protects Mice Against Diet‐Induced MASLD

To further understand the role of O‐GlcNAcylation at the T662 site in hepatocyte lipid deposition regulated by LIMA1, we examined the impact of LIMA1 T662 mutation on its function in promoting lipid deposition in hepatocytes. Overexpression of LIMA1 and LIMA1^ΔT662^ was evaluated for their effects on β‐catenin/FASn expression and lipid deposition in LIMA1 HKO MPH and HepG2^shLIMA1^ cells. Results of Western blot and Nile red staining showed that LIMA1 overexpression increased β‐catenin and FASn expression, and lipid deposition, compared to controls (Figure S7A,B,F,G, Supporting Information). Conversely, no significant differences were observed between the LIMA1 T662 site mutation group (LIMA1^ΔT662^) and controls (Figure S7A,B,F,G, Supporting Information). Co‐immunoprecipitation results indicated reduced binding of LIMA1 to β‐catenin in cells with LIMA1^ΔT662^ overexpression compared to LIMA1 overexpression (Figure S7C,H, Supporting Information). Immunofluorescence analysis revealed that LIMA1 overexpression not only enhanced β‐catenin expression but also promoted its nuclear localization in LIMA1 HKO MPH and HepG2^shLIMA1^ cells (Figure S7D,I, Supporting Information). In contrast, there were no significant differences in β‐catenin expression or nuclear localization between the LIMA1^ΔT662^ group and controls (Figure S7D,I, Supporting Information). Knockdown of LIMA1 led to decreased expression and nuclear localization of β‐catenin in cells with OPA‐induced steatosis (Figure S7E,J, Supporting Information). These results suggest that the T662 site mutation plays a critical role in LIMA1 binding to β‐catenin and in regulating β‐catenin/FASn expression and lipid deposition.

**Figure 8:**
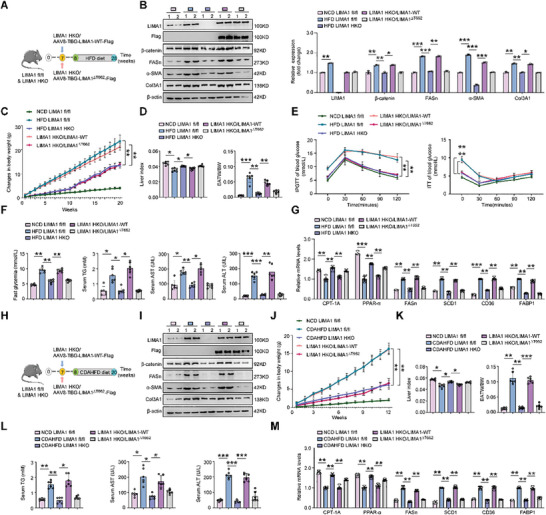
Inhibiting LIMA1 O‐GlcNAcylation protects mice against diet‐induced MASLD. A) Schematic diagram illustrating the experimental strategy. At 7 weeks of age, male LIMA1 HKO mice were injected in the tail vein with adeno‐associated virus type 8 (AAV8)‐TBG‐LIMA1‐WT‐Flag vector (LIMA1‐WT) or AAV8‐TBG‐LIMA1‐T662A‐Flag vector (LIMA1^∆T662^), and male LIMA1 fl/fl and LIMA1 HKO mice injected with AAV8‐TBG‐control vector were used as controls. After 1 week of injection, mice were fed a HFD for 20 weeks. B) Western blot analysis of LIMA1, β‐catenin, FASn, α‐SMA, and Col3A1 in livers from NCD‐fed LIMA1 fl/fl mice, HFD‐fed LIMA1 fl/fl mice, HFD‐fed LIMA1 HKO mice, HFD‐fed LIMA1 HKO mice injected AAV8‐TBG‐LIMA1‐WT‐Flag vector or AAV8‐TBG‐LIMA1‐T662A‐Flag vector. C‐F) Changes in body weight, liver index, EATW/BW, results of IPGTT and ITT assays, and fast glycemia, serum TG, serum AST, serum ALT levels in each group (*n* = 6). G) Lipometabolic mRNA expression in the liver tissues of the indicated mice (*n* = 6). H) Schematic diagram illustrating the experimental strategy. After 1 week of injection, mice were fed a CDAHFD diet for 12 weeks. I) Western blot analysis of LIMA1, β‐catenin, FASn, α‐SMA, and Col3A1 in livers from NCD‐fed LIMA1 fl/fl mice, CDAHFD‐fed LIMA1 fl/fl mice, CDAHFD‐fed LIMA1 HKO mice, CDAHFD‐fed LIMA1 HKO mice injected AAV8‐TBG‐LIMA1‐WT‐Flag vector or AAV8‐TBG‐LIMA1‐T662A‐Flag vector. J‐L) Changes in body weight, liver index, EATW/BW, and serum TG, serum AST, serum ALT levels in each group (*n* = 6). M) Lipometabolic mRNA expression in the liver tissues of the indicated mice (*n* = 6). The data were plotted as Mean ± SEM. * *p *< 0.05, ** *p* < 0.01, *** *p* < 0.001 by one‐way ANOVA.

To characterize the impact of LIMA1 O‐GlcNAcylation on MASLD progression in vivo, we constructed AAV8‐TBG vectors encoding LIMA1‐WT‐Flag and LIMA1^ΔT662^‐Flag, and injected AAV8s containing these vectors into LIMA1 HKO mice via the tail vein injection (**Figure** **8**A,H). LIMA1 fl/fl and LIMA1 HKO mice injected with control AAV8 served as controls (Figure 8A,H). After feeding HFD and CDAHFD, the expression of LIMA1, β‐catenin, FASn, α‐SMA, and Col3A1 in the liver of LIMA1 HKO mice was lower than that of LIMA1 fl/fl mice (Figure 8B,I; Figure S8A, Supporting Information). Compared with the LIMA1 fl/fl mice, the LIMA1 HKO mice exhibited lower liver weights, less weight gain of inguinal adipose tissue, and higher liver index without significantly altered of food intake (Figure 8C,D,J,K; Figure S8B,C, Supporting Information). Furthermore, the LIMA1 HKO mice exhibited marked increases in glucose tolerance and insulin sensitivity, as indicated by the IPGTT and ITT results (Figure 8E). In terms of metabolic parameters, the LIMA1 HKO mice exhibited lower fast glycemia, TG levels than those of the HFD‐fed and CDAHFD‐fed LIMA1 fl/fl mice (Figure 8F,L). Regarding liver injury markers, LIMA1 deficiency decreased the serum ALT and AST levels (Figure 8F,L). Histological analysis revealed that compared to liver sections from LIMA1 fl/fl mice, liver sections from HFD‐fed and CDAHFD‐fed LIMA1 HKO mice exhibited reduced hepatocyte death and lipid droplet staining, inflammatory cell infiltration, and hepatic fibrosis (Figure S8D,E, Supporting Information). Analysis of lipometabolic gene expression also validated the impact of LIMA1 deficiency on hepatic steatosis (Figure 8G,M). Compared with those of LIMA1 HKO/LIMA1‐WT mice, the livers of LIMA1 HKO/LIMA1^ΔT662^ mice exhibited reduced β‐catenin, FASn, α‐SMA, and Col3A1 expression (Figure 8B,I; Figure S8A, Supporting Information). Similarly, reductions in liver weight and weight gain of inguinal adipose tissue, and upregulation in liver index were also noted in the LIMA1 HKO/LIMA1^ΔT662^ mice (Figure 8C,D,J,K). In contrast, food intake was not significantly altered between different groups, and this trend was sustained over the entire treatment period (Figure S8B,C, Supporting Information). After HFD and CDAHFD diet feeding, the LIMA1 HKO/LIMA1^ΔT662^ mice exhibited systematic amelioration of MASLD‐related phenotypes, as indicated by the changes in insulin resistance (as shown by IPGTT and ITT), fast glycemia and TG contents, and serum ALT and AST levels (Figure 8E,F,L). Similarly, histopathological analysis of liver sections revealed alleviation of hepatic death and steatosis, and a reduction in inflammatory cell infiltration and fibrosis in the LIMA1 HKO/LIMA1^ΔT662^ mice compared to the LIMA1 HKO/LIMA1‐WT mice (Figure S8D,E, Supporting Information). Analysis of lipometabolic gene expression also validated the impact of LIMA1‐WT and LIMA1^ΔT662^ on hepatic steatosis (Figure 8G,M). These findings clearly indicate that inhibiting LIMA1 O‐GlcNAcylation alleviates MASLD progression.

### 2.8 LIMA1 Promoted O‐GlcNAcylation in Hepatocytes by FASn‐Mediated OGT Upregulation

**Figure 9:**
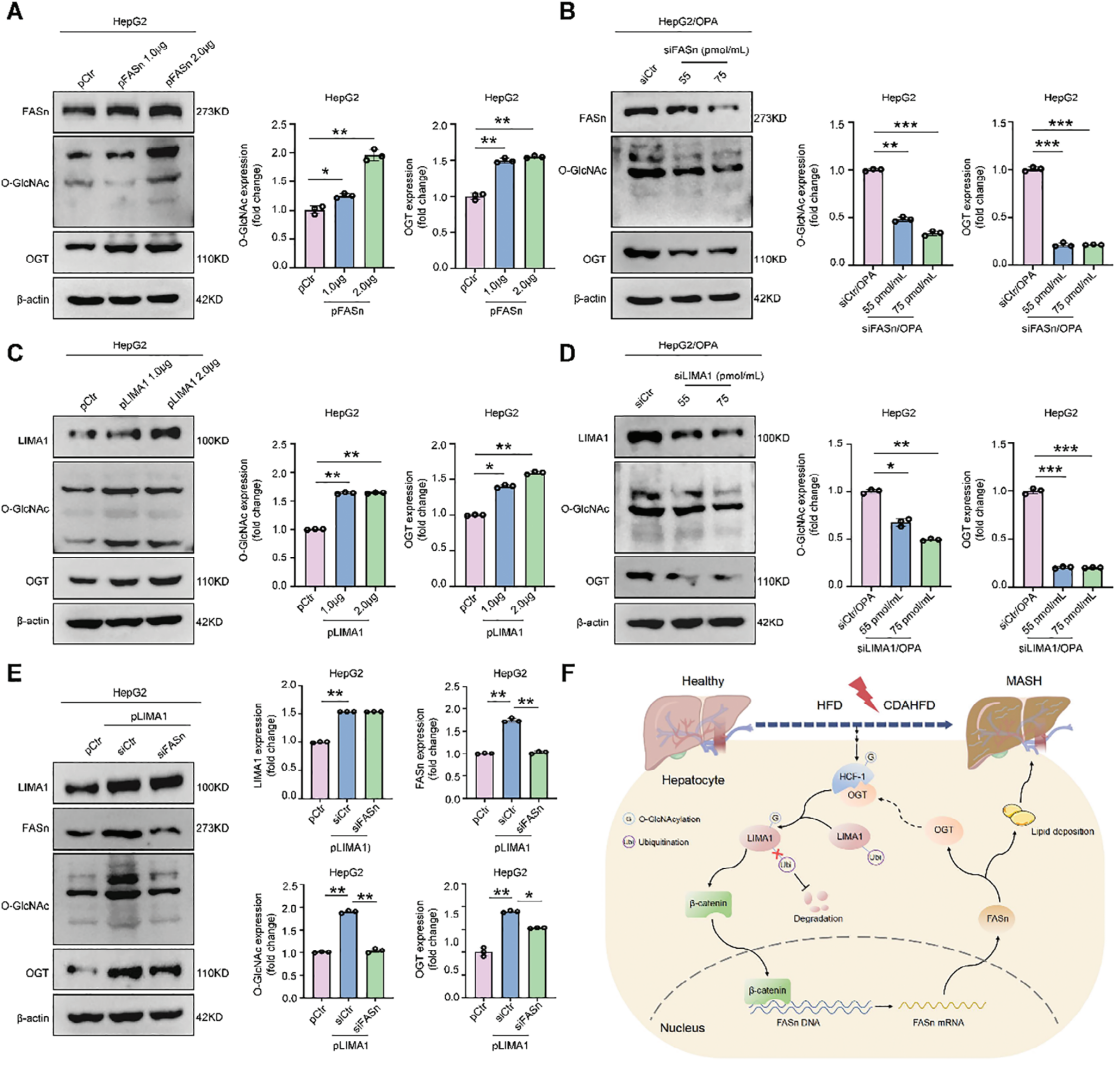
LIMA1 upregulates O‐GlcNAcylation in hepatocytes by FASn‐mediated OGT upregulation. A) Western blot analysis of FASn, O‐GlcNAc, and OGT in HepG2 cells transfected with pCtr or pFASn (*n *= 3). B) Western blot analysis of FASn, O‐GlcNAc, and OGT in OPA‐treated HepG2 cells transfected with siCtr or siFASn (*n *= 3). C) Western blot analysis of LIMA1, O‐GlcNAc, and OGT in HepG2 cells transfected with pCtr or pLIMA1 (*n *= 3). D) Western blot analysis of LIMA1, O‐GlcNAc, and OGT in OPA‐treated HepG2 cells transfected with siCtr or siLIMA1 (*n *= 3). E) Western blot analysis of LIMA1, FASn, O‐GlcNAc, and OGT in pCtr transfected HepG2 cells and pLIMA1 transfected HepG2 cells co‐transfected with siCtr or siFASn (*n *= 3). F) Schematic representation of HCF1/OGT‐induced LIMA1 O‐GlcNAcylation at T662 site regulates lipid accumulation by activating β‐catenin/FASn‐associated signaling in hepatocytes. FASn further enhances protein O‐GlcNAcylation in hepatocytes by up‐regulating OGT, thereby establishing a positive feedback loop that enables LIMA1 to increase O‐GlcNAcylation levels within hepatocytes. The data were plotted as Mean ± SEM. * *p *< 0.05, ** *p* < 0.01, *** *p* < 0.001 by one‐way ANOVA.

Previous research has shown that FASn can influence protein O‐GlcNAcylation levels by regulating OGT expression.^[^ ^24^ ^]^ Then we explored the role of LIMA1 in modulating protein O‐GlcNAcylation in hepatocytes via FASn. The results demonstrated that increased FASn expression led to elevated O‐GlcNAc and OGT levels in normal HepG2 cells (**Figure** **9**A; Figure S8F, Supporting Information). Conversely, in OPA‐damaged HepG2 cells, knocking down FASn resulted in decreased O‐GlcNAc and OGT levels (Figure 9B; Figure S8G, Supporting Information). These findings suggest that FASn can impact cellular responses to lipotoxic damage by altering O‐GlcNAcylation patterns. Additionally, overexpression of LIMA1 also influenced O‐GlcNAc and OGT expression in normal HepG2 cells (Figure 9C; Figure S8H, Supporting Information), with opposite effects observed in OPA‐damaged HepG2 cells upon LIMA1 knockdown (Figure 9D; Figure S8I, Supporting Information). Interestingly, knocking down FASn attenuated the effects of LIMA1 overexpression on O‐GlcNAc and OGT expression levels (Figure 9E). The study revealed that steatosis upregulated HCF1 and OGT expression, leading to catalyzed O‐GlcNAcylation of LIMA1 protein and subsequent inhibition of its ubiquitin‐dependent degradation. Subsequently, O‐GlcNAcylation of LIMA1 activates β‐catenin/FASn‐related pathway, causing lipid accumulation in hepatocytes. FASn further enhances protein O‐GlcNAcylation in hepatocytes by up‐regulating OGT, thereby establishing a positive feedback loop that enables LIMA1 to increase O‐GlcNAcylation levels within hepatocytes (Figure 9F).

### 2.9 LIMA1 Knockdown Reduces LTH‐sEV Promoted MASLD Progression and HSC Activation

Lipotoxic hepatocytes‐derived small extracellular vesicles (LTH‐sEV) can promote the progression of MASLD by transporting active factors. In HFD‐fed and CDAHFD‐fed mice injected with BAGN, there was a decrease in circulating sEV levels (Figure S9A, Supporting Information). Furthermore, BAGN treatment resulted in a reduction of sEV levels derived from OPA‐induced steatotic MPH and HepG2 cells (Figure S9B, Supporting Information). In addition, NGT‐induced O‐GlcNAcylation in MPH and HepG2 cells was associated with an increase in sEV levels (Figure S9C, Supporting Information). To prove that LIMA1 is an essential active molecule in LTH‐sEV, we observed the effect of LIMA1 knockdown in LTH‐sEV (sEV^shLIMA1^) on MASLD progression both in vitro and in vivo. Compared to LTH‐sEV with control shRNA (sEV^shCtr^), the level of LIMA1 in sEV^shLIMA1^ was decreased, and there was no significant difference in diameter and CD63 expression (Figure S9D,E, Supporting Information). Importantly, they did not express the endoplasmic reticulum protein Calnexin (Figure S9D, Supporting Information). After injection into HFD‐fed mice, CD9 staining showed that both sEV^shCtr^ and sEV^shLIMA1^ could localize in hepatic cells in HFD‐fed mice (Figure S9F,G, Supporting Information). sEV^shCtr^ significantly increased hepatic LIMA1 expression in HFD‐fed mice, which was not found in sEV^shLIMA1^ treated mice (Figure S9G, Supporting Information). Oil Red staining showed that compared with sEV^shLIMA1^, sEV^shCtr^ had a more significant effect on promoting vacuolar degeneration and fat deposition of hepatocytes in injured liver tissue (Figure S9H, Supporting Information). In HFD‐fed mice, sEV^shCtr^ promoted the expression of macrophages marker F4/80 and inflammatory factor IL‐6, and decreased the expression of anti‐inflammatory factors IL‐10, but abolished by LIMA1 knockdown in LTH‐sEV (Figure S9I,J, Supporting Information). Morever, sEV^shCtr^ promoted collagen deposition, the protein expression of α‐SMA, and the mRNA expression of Col1A2 and Col3A1 in liver tissue (Figure S9K–M, Supporting Information). Enhanced hepatic α‐SMA, Col1A2, Col3A1 expression, and collagen deposition were not found in sEV^shLIMA1^‐treated mice. Additionally, liver function indicators revealed that serum TG, AST, and ALT were significantly increased in HFD‐fed mice injected with sEV^shCtr^ compared with mice injected with sEV^shLIMA1^ (Figure S9N, Supporting Information). Immunohistochemistry and Western blot analysis demonstrated that sEV^shCtr^ increased the expression of β‐catenin and FASn, but not in sEV^shLIMA1^‐treated mice (Figure S9O,P, Supporting Information).

HSC activation plays a vital role in the progression of MASLD to liver fibrosis.^[^ ^25^ ^]^ Thus, we then explored the regulatory role of LIMA1 derived from LTH‐sEV on HSC activation in vitro. Compared with PBS control, sEV^shCtr^ could upregulate the protein expression of LIMA1 and α‐SMA, and mRNA expression of Col3A1 and Col1A2 in LX‐2 cells (Figure S10A–C, Supporting Information). However, sEV^shLIMA1^ significantly reduced the promotion of LIMA1 protein, α‐SMA protein, Col3A1 mRNA, and Col1A2 mRNA expression (Figure S10A–C, Supporting Information). We also found that the expression of α‐SMA protein, Col3A1 mRNA, and Col1A2 mRNA in LX‐2 cells could be up‐regulated by pLIMA1 transfection and decreased by LIMA1 knockdown (Figure S10D–I, Supporting Information). In contrast, LIMA1 expression was unaltered in LX2 cells after TGF‐β treatment (Figure S10J, Supporting Information). These results suggest that LTH‐sEV mediated delivery of LIMA1 promoted MASLD progression by promoting HSC activation (Figure S10K, Supporting Information).

## 3 Discussion

Early diagnosis and treatment of metabolic dysfunction‐associated steatotic liver disease (MASLD) remains a significant challenge.^[^ ^26^ ^]^ Current research is focused on understanding the mechanisms of lipid metabolism in hepatocytes within the context of MASLD.^[^ ^27^ ^]^ This study has unveiled a new mechanism involving LIMA1 in hepatocyte lipid deposition and MASLD progression. The results indicate that detecting serum sEV LIMA1 demonstrates high sensitivity and specificity in diagnosing metabolic‐dysfunction‐associated steatohepatitis (MASH). Furthermore, a novel mechanism was discovered where the combined action of HCF1/OGT promotes LIMA1 O‐GlcNAcylation at the T662 site, preventing its ubiquitination and degradation in steatotic hepatocytes. The study also reveals that O‐GlcNAcylation of LIMA1 enhances hepatocyte lipid deposition by activating β‐catenin/FASn‐associated signaling. Inhibiting LIMA1 O‐GlcNAcylation protects mice against diet‐induced MASLD. Importantly, it was confirmed that LIMA1 upregulates O‐GlcNAcylation in hepatocytes by modulating FASn to increase OGT expression. Morever, LTH‐sEV‐mediated delivery of LIMA1 promoted MASLD progression by promoting HSC activation.

LIMA1, a crucial regulator of the actin cytoskeleton, is initially identified as a frequently downregulated tumor suppressor in epithelial tumors.^[^ ^15^ ^]^ LIMA1 deficiency can disrupt cytoskeletal dynamics, affect cell motility, and impair intercellular adhesion, ultimately promoting tumor growth, invasion, and migration.^[^ ^28^ ^]^ Additionally, LIMA1 plays a vital role in regulating intestinal cholesterol absorption.^[^ ^17^ ^]^ However, its involvement in hepatocyte lipid metabolism and MASLD progression remains unclear. Our findings suggest that elevated levels of serum LIMA1 may indicate liver damage in MASH patients. We also observed a significant increase in LIMA1 protein levels in liver tissues from HFD‐fed and CDAHFD‐fed mice, and steatotic hepatocytes. Furthermore, serum LIMA1 levels was upregulated in HFD‐fed and CDAHFD‐fed LIMA1 fl/fl mice, but downregulated in LIMA1 HKO mice, indicating that hepatocytes secreted LIMA1 into mouse serum. Notably, LIMA1 HKO exhibited systematic amelioration of MASLD‐related phenotypes, as indicated by the changes in hepatic steatosis, inflammatory cell infiltration, and hepatic fibrosis in HFD‐fed and CDAHFD‐fed mice. Moreover, inhibiting LIMA1 O‐GlcNAcylation protects mice against diet‐induced MASLD. These results offer compelling evidence of the relationship between LIMA1 and hepatocyte lipid metabolism and MASLD progression.

O‐GlcNAcylation is a crucial post‐translational modification that regulate protein function and its impact on hepatocyte lipid deposition. Previous studies have shown that O‐GlcNAcylation levels increase in the livers of both human and animal models with steatohepatitiss.^[^ ^29^ ^]^ Consuming a high‐fat diet has been linked to increased O‐GlcNAcylation. This, in turn, is responsible for disrupting the AMPK/ACC pathway,^[^ ^11^ ^]^ leading to excessive accumulation of lipids. Moreover, O‐GlcNAcylation of SREBP‐1 plays a crucial role in changing hepatic lipid synthesis and the progression of fatty liver disease.^[^ ^30^ ^]^ Inhibition of O‐GlcNAcylation of nuclear factor‐κB (NF‐κB) has been found to reduce hepatic steatosis.^[^ ^31^ ^]^ Our research aligns with these findings, demonstrating that inhibiting O‐GlcNAcylation with BAGN effectively inhibits lipid accumulation, inflammation, and collagen deposition, and promotes liver function recovery in HFD‐fed and CDAHFD‐fed mice. However, further investigation is needed to identify the specific target proteins that undergo O‐GlcNAcylation and the mechanisms by which they regulate MASLD. Previous studies have suggested that HCF1 promotes O‐GlcNAcylation of PGC1α and NRF1 by recruiting OGT, acting as a central regulator of transcription.^[^ ^20^, ^21^ ^]^ Nevertheless, the regulation of protein O‐GlcNAcylation by the HCF1/OGT complex in hepatocytes remains unexplored. In our study, we found that increased HCF1 in steatotic hepatocytes recruited OGT and induced O‐GlcNAcylation at the T662 site of LIMA1 protein, providing insight into the regulatory mechanism of O‐GlcNAcylation in steatotic hepatocytes.

Protein O‐GlcNAcylation and ubiquitination are both post‐translational modifications that interact to regulate protein activity.^[^ ^32^ ^]^ In hepatocellular carcinoma (HCC), O‐GlcNAcylation of Thr 168 and Thr 177 of eIF4E protects it from ubiquitin‐proteasome‐mediated protein degradation, promoting the stem‐like cell potential of liver cancer cells.^[^ ^33^ ^]^ In hepatocytes of MASH, O‐GlcNAcylation influences FASn expression by protecting FASn from ubiquitination degradation, enhancing FASn activity, and hepatic lipid deposition under high‐fat conditions.^[^ ^34^ ^]^ Our research findings demonstrate that O‐GlcNAcylation at the T662 site of LIMA1 protein inhibits the ubiquitination degradation of LIMA1, thereby increasing its stability.

Hepatic FASn expression is significantly increased in patients with MASLD.^[^ ^35^ ^]^ FASn plays a crucial role in hepatic de novo lipogenesis (DNL) and contributes to triglyceride (TG) accumulation in the liver.^[^ ^36^ ^]^ Due to its strong lipogenic capacity, FASn is considered a promising target for treating metabolic diseases. Several transcription factors, including sterol‐regulatory element‐binding protein‐1 (SREBP‐1) and carbohydrate receptor element‐binding proteins (ChREBP), have been identified as regulators of FASn expression.^[^ ^37^, ^38^ ^]^ LIMA1, also known as SREBP‐3, can induce lipid deposition in hepatocytes by modulating FASn expression, despite lacking transcriptional activity.^[^ ^17^ ^]^ β‐catenin, a critical protein involved in lipid deposition, is linked to the progression of metabolic dysfunction‐associated steatotic liver (MASL) to MASH, liver fibrosis, and HCC.^[^ ^39^, ^40^, ^41^ ^]^ Aberrant activation of the Wnt/β‐catenin signaling pathway can enhance lipid synthesis by FASn in hepatocytes in MASLD.^[^ ^22^, ^42^ ^]^ This study validates the role of β‐catenin in LIMA1‐mediated regulation of FASn expression to promote lipid deposition in MASLD. Recent evidence suggests that FASn can influence protein O‐GlcNAcylation levels by regulating OGT expression.^[^ ^24^ ^]^ Similarly, our findings demonstrate that FASn can enhance protein O‐GlcNAcylation by up‐regulating OGT in steatotic hepatocytes. Interestingly, LIMA1 enhances O‐GlcNAcylation in steatotic hepatocytes through FASn‐mediated OGT upregulation.

Recently, small extracellular vesicles (sEV) and their contents have emerged as potential biomarkers for liver disease.^[^ ^43^ ^]^ Numerous studies have explored the role of lipotoxic hepatocyte‐derived sEV in the initiation and progression of MASLD.^[^ ^44^, ^45^ ^]^ It was also discovered that altered protein expression in sEV could act as messenger molecules to promote the progression of MASLD by transmitting signals between cells. The researchers conducted a proteomic analysis of sEV from N‐sEV and lipotoxic MPH‐sEV using LC‐MS/MS to investigate this further. Our study revealed a significant increase in the protein level of LIMA1 in lipotoxic MPH‐sEV. The study also found that elevated levels of LIMA1‐containing serum sEV may indicate liver damage in MASH patients. Activating HSC plays a critical role in progressing MASLD to fibrosis.^[^ ^25^ ^]^ In this study, we observed a significant increase in LIMA1 protein levels in activating HSC within the liver tissue of HFD‐fed and CDAHFD‐fed mice. Additionally, the study revealed that LTH‐sEV‐mediated delivery of LIMA1 promoted the progression of MASLD by promoting HSC activation.

This study has several limitations. First, we focused on the role of O‐GlcNAcylation in regulating LIMA1 protein stability without investigating the impact of other post‐translational modifications, such as phosphorylation and acetylation on LIMA1. Furthermore, LIMA1 may regulate lipid deposition through alternative signaling pathway molecules besides the β‐catenin protein pathway. To deepen our understanding of LIMA1's role and mechanism in hepatocyte lipid deposition, we intend to conduct a multi‐omics analysis to observe the impact of LIMA1 on lipid metabolism and related signaling pathways. This analysis will help clarify the regulatory effect of LIMA1 on key molecules and elucidate the mechanism by which LIMA1 inhibits lipid deposition in hepatocytes.

Our study indicates that serum sEV LIMA1 may serve as a noninvasive biomarker for MASH. The HCF1/OGT complex induces O‐GlcNAcylation of the LIMA1 protein, preventing its ubiquitination and degradation. This O‐GlcNAcylation of LIMA1 promotes lipid accumulation in hepatocytes by activating the β‐catenin/FASn signaling pathway. Inhibiting LIMA1 O‐GlcNAcylation protects mice against diet‐induced MASLD. Furthermore, elevated O‐GlcNAcylation levels were observed in LIMA1‐upregulated hepatocytes due to FASn‐mediated OGT upregulation. Moreover, LTH‐sEV‐mediated delivery of LIMA1 promoted MASLD progression by promoting HSC activation. This investigation deepens our understanding of the role of LIMA1 in the progression of MASLD and proposes a potential therapeutic strategy for MASH by targeting LIMA1 O‐GlcNAcylation.

## 4 Experimental Section

### Cell Culture

Human liver hepatocellular carcinoma cell line HepG2, human hepatic stellate cell LX2, and HEK293T cells were obtained from Fenghui Biology Co., Ltd (Hunan, China). HepG2 cells, LX2 cells, and HEK293T cells were cultured in DMEM/high glucose with 10% FBS (Bio‐channel, Nanjing, China, BC‐SE‐FBS07) and 100 U penicillin/streptomycin (Invitrogen, USA, 15 140 148). Mouse primary hepatocytes (MPH) were acquired from C57BL/6 male mice aged 6–8 weeks using collagenase perfusion and gradient centrifugation.^[^ ^46^ ^]^ In brief, the isolated cells were the filtered through a 100 µm cell strainer and were plated in collagen I‐coated well plated in DMEM containing 10% FBS and 1% penicillin/streptomycin. All cells were maintained at 37 °C in a humidified atmosphere with 5% CO2. To induce steatosis, HepG2 cells and MPH were treated with a OPA mixture (0.001 м) for 24 h.^[^ ^47^ ^]^ The OPA mixture consisted of oleic acid (C18:1) (Sigma, USA, O7501) and palmitic acid (C16:0) (Sigma, USA, P9767) at a ratio of 2:1 dissolved in a 5% BSA solution (BioFroxx, Germany, 4240).

### Small Extracellular Vesicles (sEV) Isolation

Mouse primary hepatocyte‐derived small extracellular vesicles (N‐sEV), lipotoxic mouse primary hepatocyte‐derived sEV (MPH‐sEV), lipotoxic HepG2 knockdown with LIMA1‐derived sEV (LTH‐sEV^shLIMA1^), and serum sEV from patients with MASH and healthy controls were isolated through ultracentrifugation.^[^ ^48^ ^]^ Briefly, Lentiviral vector containing LIMA1 shRNA sequence (pLKO.1‐U6‐L02‐hPGK‐copGFP‐Puro‐shLIMA1; Vigen Biotech, China) (sequence: GTC TCT GAA TTG GTC GAG TTT) was designed for explicitly targeting LIMA1 silencing in HepG2 cells; a negative control vector (pLKO.1‐Puro‐shRNA) was also prepared. OPA treated MPH, HepG2^shCtr^ cells, and HepG2^shLIMA1^ cells were cultured in DMEM/high glucose or DMEM medium comprising 10% FBS for 24 h, respectively. When the cells reached 70% confluence, the media of cells was changed to an FBS‐free medium for 24 h; the conditioned medium was collected and centrifuged at 300 × g for 10 min, at 2000 × g for 10 min, and at 10000 × g for 30 min to remove cells and cell debris. The concentrated cell culture supernatant and serum underwent ultracentrifugation at 100000 × g for 70 min using an Optima L‐90K centrifuge (Beckman Coulter, Brea, CA). The sEV‐enriched fraction was collected at the bottom of the tube, washed 3 times with phosphate‐buffered saline (PBS) by centrifugation at 1500 × g for 30 min with a 100‐KDa MWCO filter, and then passed through a 0.22‐mm filter before being stored at ‐80 °C. Specific expressions of  ALIX (Proteintech, Wuhan, China, 12422‐1‐AP), TSG101 (Proteintech, Wuhan, China, 28283‐1‐AP), and CD9 (Cell Signaling Technology, USA, 98327S), Calnexin (Proteintech, Wuhan, China, 10427‐2‐AP) was assessed via western blot. The morphologies and size distribution of sEV were analyzed using transmission electron microscopy (TEM, FEI Tecnai 12, Philips, The Netherlands) and nanoparticle tracking analysis (NTA, ZetaView, Germany).

### Western Blotting

Protein was extracted from liver tissues and cultured cells using RIPA (Beyotime, Shanghai, China, P0013B). The concentrations of protein samples were determined by BCA assay (Beyotime, Shanghai, China, P0009). Proteins were separated on a 10% SDS‐PAGE denaturing gel and transferred to the PVDF membrane. Next, the membrane was blocked with 5% milk at room temperature for 1 h. The membrane with blotted protein was incubated with primary antibodies against β‐actin (Abclonal, Wuhan, China, AC006), LIMA1 (Proteintech, Wuhan, China, 66071‐1‐Ig), O‐GlcNAc (Cell Signaling Technology, USA, 9875S), β‐catenin (Proteintech, Wuhan, China, 17565‐1‐AP), FASn (Proteintech, Wuhan, China, 10624‐2‐AP), OGT (Proteintech, Wuhan, China, 66823‐1‐Ig), OGA (Proteintech, Wuhan, China, 14711‐1‐AP), HCF1 (Cell Signaling Technology, USA, 50708S), DYKDDDDK tag (Proteintech, Wuhan, China, 66008‐4‐Ig), MYC tag (Proteintech, Wuhan, China, 60003‐2‐Ig), His tag (Proteintech, Wuhan, China, 66005‐1‐Ig), HA tag (Proteintech, Wuhan, China, 51064‐2‐AP), ALIX (Proteintech, Wuhan, China, 12422‐1‐AP), TSG101 (Proteintech, Wuhan, China, 28283‐1‐AP), CD9 (Cell Signaling Technology, USA, 98327S), CD63 (Cell Signaling Technology, USA, 52090S), Calnexin (Proteintech, Wuhan, China, 10427‐2‐AP), α‐SMA (Proteintech, Wuhan, China, 55135‐1‐AP), and Col3A1 (Proteintech, Wuhan, China, 22734‐1‐AP). Following this, the membrane was probed with horseradish peroxidase‐conjugated secondary antibodies (Invitrogen, USA, 31 460) and the protein bands were visualized using enhanced chemiluminescence reagents (Vazyme, Nanjing, China, E412‐01).

### Liquid Chromatography‐Tandem Mass Spectrometry (LC‐MS/MS)

N‐sEV and MPH‐sEV were re‐suspended in PBS (50 µL), Triton X‐100 (2 µL), and PMSF (5 µL) to dissolve the vesicles. The insoluble fraction was pelleted by centrifugation at 20000 × g. Subsequently, the insoluble fraction was acetone‐precipitated at ‐20 °C, followed by digestion in gel with trypsin (sequencing grade, Promega) for 18 h at 37 °C. The resulting peptides were analyzed by LC‐MS/MS on a Q‐Exactive‐Orbitrap mass spectrometer (Thermo Scientific, Waltham, MA, USA). The fold change represents the ratio of direct reporter group strength, with a difference threshold of 1.13 times and a significance level of *p* < 0.05. Gene Denovo Biotechnology Co., Ltd, Guangzhou, conducted all sample sequencing analyses.

### Animal Models

C57BL/6 male mice aged 6–8 weeks were included in this study, and housed in pathogen‐free conditions with a 12 h light/dark cycle and temperature kept at 22–24 °C, humidity kept at 40%–70%. To establish a metabolic dysfunction‐associated steatotic liver (MASL) model, mice were fed with a high‐fat diet (HFD; protein, 20%; fat, 60%; carbohydrates, 20%; H10060; HUAFUKANG Bioscience; Beijing, China) for 20 weeks. To establish a metabolic dysfunction‐associated steatohepatitis (MASH) model, mice were fed with a choline‐deficient, L‐amino acid‐defined, high‐fat diet (CDAHFD; A06071302; Research diet, New Brunswick, NJ, USA) for 12 weeks. The mice in the control group were fed with a normal chow diet (NCD; protein, 18.3%; fat, 10.2%; carbohydrates, 71.5%; 1 010 001; XIETONG BIO‐ENGINEERING; Jiangsu, China) for corresponding time durations. All experiments involving animals were conducted according to the ethical policies and procedures approved by the Jiangsu University ethics committee (Approval no. UJS‐IACUC‐AP‐2020033127).

### Oil Red O Staining

Liver samples were frozen in optimum cutting temperature compound OCT and sectioned. The sections of each group were dyed with Oil Red O (Sigma, USA, O0625) to detect neutral lipid deposition following standard procedures. Tissue slices were stained with 5% Oil Red O (in 60% isopropanol) at room temperature for 10 min. Oil droplet images were observed using a vertical fluorescence microscope (Nikon TE300, Japan).

### Immunofluorescence

Liver tissue slides and cultured cells were fixed in 4% paraformaldehyde for 30 min at 4 °C and then ruptured with 0.1% Triton X‐100 (dilution in PBS) and blocked with 5% BSA. Liver tissue slides and cultured cells were incubated with anti‐LIMA1 (Proteintech, Wuhan, China, 66071‐1‐Ig), anti‐O‐GlcNAc (Cell Signaling Technology, USA, 9875S), anti‐β‐catenin (Proteintech, Wuhan, China, 17565‐1‐AP), anti‐HCF1 (Cell Signaling Technology, USA, 50708S), anti‐OGT (Proteintech, Wuhan, China, 66823‐1‐Ig), anti‐F4/80 (Proteintech, Wuhan, China, 28463‐1‐AP), anti‐α‐SMA (Proteintech, Wuhan, China, 55135‐1‐AP), anti‐CD9 (Cell Signaling Technology, USA, 98327S), and anti‐CD63 (Cell Signaling Technology, USA, 52090S). Then, the liver tissue slides and cultured cells were labeled with FITC‐conjugated goat anti‐Rabbit IgG (Abclonal, Wuhan, China, AS019) and Cy3‐conjugated Goat anti‐Mouse IgG (Abclonal, Wuhan, China, AS008). The nuclei were stained with Hoechst33342 (Sigma, USA, H21842). The slides were observed with a confocal microscope (DeltaVision Elite, USA).

### Masson Staining

The liver tissues were removed and fixed in 4% paraformaldehyde solution for 24 h, and then dehydrated and embedded with gradient ethanol and sectioned. The liver tissue slides of mice in each group were stained by Masson staining (Solarbio, Beijing, China, G1346) according to the manufacturer's instructions.

### Immunohistochemistry

The liver tissue slides underwent deparaffinization and rehydration for immunohistochemical staining of LIMA1 (Proteintech, Wuhan, China, 66071‐1‐Ig), β‐catenin (Proteintech, Wuhan, China, 17565‐1‐AP), FASn (Proteintech, Wuhan, China, 10624‐2‐AP), and α‐SMA (Proteintech, Wuhan, China, 55135‐1‐AP). Endogenous peroxidase activity was inhibited using a 3% hydrogen peroxide (H_2_O_2_) solution for 30 min. Subsequently, the slides were treated with preheated antigen retrieval solution (0.01 м, pH 6.0, citrate buffer) for another 30 min. Following blocking with 5% BSA, the slides were incubated overnight at 4 °C with a primary antibody against LIMA1, β‐catenin, FASn, and α‐SMA. This was followed by incubation with biotin‐conjugated anti‐rabbit IgG and streptavidin‐biotin (Boster, Wuhan, China, SA1020). Finally, the liver tissue slides were visualized using the DAB Horseradish Peroxidase Color Development Kit (Boster, Wuhan, China, AR1022) and counterstained with hematoxylin. Image acquisition was performed using a pathological section scanner (3DHISTECH, Hungary).

### Human Serum Samples

Informed consent was obtained from all subjects and approved by the Ethics Committee of Jiangsu University (2 012 258). Blood samples were collected from 50 MASH patients and 50 healthy volunteers at the Third People's Hospital of Changzhou, China, following informed consent. The serum was isolated from the samples through centrifugation and stored at ‐80 °C until analysis. Pathological examination confirmed all samples and histological grade was determined based on the World Health Organization (WHO) classification. Characteristics of male and female MASH patients used in this study at Table S1 (Supporting Information).

### Enzyme‐Linked Immunosorbent Assay (ELISA)

Following the manufacturer's instructions, serum and serum‐derived sEV lysates (400 µg mL^−1^) were added to the wells of an ELISA microtiter plate. The plate was then incubated for 2 h at 37 °C, followed by an additional 1 h incubation at room temperature with primary antibodies, specifically anti‐LIMA1 antibodies at a 1:100 dilution for sEV lysates. Subsequently, the samples were washed three times and incubated with anti‐rabbit HRP‐conjugated secondary antibody at a 1:5000 dilution for 1 h at room temperature. After another three washes, tetramethyl‐benzidine substrates were added to the samples treated with the secondary antibody and incubated in darkness for 1 h at room temperature. Following three final washes, the absorbance at 450 nm was measured using a microplate reader within 2 min of adding the “Stop Solution” to the wells. The control consisted of the absorbance value obtained from a well without any sample. The ELISA kit utilized in this experiment was the LIM Domain And Actin‐Binding Protein 1 (LIMA1) ELISA Kit from Abbexa (UK; Human) and MyBioSource (UK; Mouse), with the catalog number abx259562 and MBS2890188.

### LIMA1 O‐GlcNAcylation Site Mapping

Liquid chromatography‐tandem mass spectrometry (LC‐MS/MS) was performed to identify the LIMA1 O‐GlcNAcylation sites, as previously described.^[^ ^49^ ^]^ In brief, human hepatocytes were treated with oleic acid and palmitic acid, and then LIMA1 was immunoprecipitated and stained with Coomassie blue. A gel containing LIMA1 was excised and entrusted to the Bio‐Tech Pack Technology Co., Ltd (Beijing, China) for analysis.

### Plasmid and siRNA Constructs and LIMA1 Overexpression Adenovirus

The full‐length cDNA of Human LIMA1 (NM_0 011 13546.2), OGT (NM_181 672.3), HCF1 (NM_0 014 10705.1), and FASn (NM_0 04104.5) were amplified by PCR and subcloned into the pcDNA3.1‐3×Flag or pcDNA3.1‐MYC or pcDNA3.1‐His. Flag‐tagged LIMA1^∆T662^ was constructed by overlapping PCR. Ubiquitin‐HA plasmids were from Fenghui Biotechnology Co., Ltd (Hunan, China). Small interfering RNA (siRNA) targeting LIMA1, HCF1, FASn, and a control scrambled siRNA were designed by Genepharma Biotechnology Co., Ltd (Suzhou, China). Mus‐LIMA1 overexpression adenovirus (pADV‐CMV‐LIMA1‐Flag) were obtained from Weizhen biosciences (Shandong, China).

### Co‐Immunoprecipitation (Co‐IP) Assay

For endogenous Co‐IP assays, MPH or HepG2 cells were divided into two groups: the untreated group (OPA 0 h) and the group subjected to oleate and palmitate stimulation for 24 h (OPA 24 h). The cells in both groups were immunoprecipitated with the indicated primary antibody. The beads were then washed with NaCl buffer and boiled with 2×SDS loading buffer before analysis by Western blot. For exogenous Co‐IP, HepG2 cells or HEK293T cells were transfected with Flag‐tagged LIMA1 or Flag‐tagged LIMA1^∆T662^ (T662A mutant) or HA‐Ubiquitin (HA‐Ubi) or MYC‐tagged HCF1 or His‐tagged OGT or si‐HCF1. Pre‐cleared cell lysates were incubated with anti‐Flag antibody or anti‐MYC overnight at 4 °C and subsequently incubated with protein A/G beads (Beyotime, Shanghai, China, P2179M) for 4 h. Immunoprecipitants were washed and immunoblotted with the indicated antibodies, respectively.

### Cycloheximide Chase Assay

MPH or HEK293T cells expressing various constructs indicated in the figures were incubated with cycloheximide (20 µg mL^−1^; CHX, MCE, USA, HY‐12320) or cycloheximide (50 µg mL^−1^) at 37 °C, respectively. At times displayed in the figures, cells were harvested, and lysates were prepared for immunoblotting.

### In Vitro Ubiquitination Assay

Cells were transfected with Flag‐tagged LIMA1 or Flag‐tagged LIMA1^∆T662^ (T662A mutant), HA‐Ubiquitin (HA‐Ubi), MYC‐tagged HCF1, or His‐tagged OGT. To prevent degradation, MG132 (10 μм) was applied before harvesting. After 48 h post‐transfection, cells were harvested using a lysis buffer containing 1% SDS and boiled for denaturation. Immunoprecipitation with anti‐Flag antibodies was performed, followed by incubation with protein A/G beads for 4 h. The captured complexes on beads were washed four times and analyzed by western blotting. The levels of LIMA1 ubiquitination were examined using anti‐HA antibodies.

### Quantitative Reverse Transcription PCR (qRT‐PCR)

The total RNA of cultured cells and liver tissues was isolated with TRIzol reagent (Invitrogen, USA, 15596026CN), and 1 mg RNA was reverse transcribed to cDNA with RNeasy kit (QIAGEN, Germany, 215 011). Quantitative RT‐PCR (qRT‐PCR) was carried out using the Evo M‐MLV RT Premix (Accurate Biology, China), the relative mRNA expression was measured by the 2^−ΔΔCt^ method, and β‐Actin was used as a control. The primers used in this study at Table S2 (Supporting Information).

### Nile Red Staining

Nile Red (1 mg mL^−1^) (Sigma, USA, 72 485) was prepared in acetone. To visualize lipid droplets, 4% paraformaldehyde‐fixed cells were dyed with Nile red (1:3000 dilution) for 5 min, followed by PBS washing and Hoechst33342 staining. Fluorescence was determined by CytationTM5 (BioTek, USA) and quantified by Image‐J 7.0 software.

### Benzyl‐α‐GalNAc (BAGN) Injection

Established HFD‐fed mice and CDAHFD‐fed mice were randomly assigned into three groups, respectively. The groups included mice fed a normal chow diet with 10% kcal from fat (NCD group, *n* = 6, respectively), mice fed a high‐fat diet (HFD group, *n* = 6), mice fed a choline‐deficient, L‐amino acid‐defined, high‐fat diet (CDAHFD group, *n* = 6), HFD‐fed mice injected with Benzyl‐α‐GalNAc (MCE, USA, HY‐ 129 389), and CDAHFD‐fed mice injected with Benzyl‐α‐GalNAc via tail vein twice a week for a duration of 4 weeks (BAGN, 25 mg kg^−1^, *n* = 6, respectively). Body weight was monitored every week throughout the experiment. Mice (*n* = 6 in each group) were individually housed and cumulative food intake in these animals was calculated as the difference between the amount of food given and the amount of food that remained every week. All animals were euthanized after overnight fasting (12 h) at the end of the study. Blood samples collected from the retinal vein plexus were centrifuged (3500 rpm, 10 min, 4 °C) to separate the serum and then stored at −80 °C for further research. To calculate the liver index (liver weight/body weight) and EATW/BW (relative weight of epididymal adipose tissue to body weight), the entire liver and epididymal adipose tissue was removed and weighed.

### Glucose and Insulin Tolerance Tests

Blood glucose levels were monitored at time points as indicated. For the insulin tolerance test (ITT), mice fasted for 6 h, and insulin (0.75 units kg^−1^ Humulin R; Eli Lilly, Indianapolis, IN, USA) was administered intraperitoneally. The intraperitoneal glucose tolerance test (IPGTT) was performed at the end of the study; mice fasted for 16 h, and glucose was then injected intraperitoneally (1 g kg^−1^ body weight). The AUC of glucose was calculated during the course of the tests.

### Analysis of Serum Biochemical Indicators

The serum levels of Triglyceride (TG), alanine transaminase (ALT), and aspartate transaminase (AST) in mice were detected according to manufacturers' instructions with a MINDRAY BS200 chemistry analyzer (MINDRAY Medical International Co., Shenzhen, China).

### Tunel Staining

Liver tissue slides were fixed in 4% paraformaldehyde for 20 min and washed with PBS for 5 min. Subsequently, the liver tissue slides were stained with a Tunel reaction mixture (Vazyme, Nanjing, China, A111‐01) for 60 min at 37 °C, following a PBS wash. Finally, the cell nuclei were stained with Hoechst33342 and observed using fluorescence confocal laser microscopy.

### Generation of Genetically Modified Mice

Hepatic LIMA1 knockout mice (LIMA1 HKO, Strain NO. T044132)were purchased from GemPharmatech (Nanjing, China). LIMA1 HKO mice was generated using the CRISPR/Cas9 system and Cre‐loxP‐mediated recombination technology. First, two single guide RNAs (sgRNA1 and sgRNA2) were used to target a fragment of LIMA1 exon3. Cas9 mRNA and gRNA were obtained by in vitro transcription. The donor vector was constructed by in‐fusion cloning, which contained a 3.0 kb 5′ homology arm, a 1.0 kb flox region and a 3.0 kb 3′ homology arm. Microinjection of Cas9 mRNA, gRNA, and donor vector into zygotes of C57BL/6 mice. One founder mouse was selected and crossed it with a C57BL/6 mouse to generate LIMA1‐Flox mice. Finally, the LIMA1 HKO mice were generated by crossing albumin‐Cre recombinase (Alb‐Cre) transgenic mice with LIMA1 flox/flox mice. Littermates Alb‐Cre negative, LIMA1 flox/flox mice (LIMA1 fl/fl) were used as controls. Mice were housed in barrier facility with free access to drinking water and fed standard chow diet.

To generate hepatic LIMA1 overexpression mice, AAV8‐TBG‐LIMA1‐WT‐Flag (LIMA1‐WT) and AAV8‐TBG‐LIMA1‐T662A‐Flag adenoviruses (LIMA1^∆T662^) were injected intravenously by LIMA1 HKO mice tail at 5 × 10^11^ vg per mice, single injection. The control LIMA1 HKO mice were injected with AAV8‐TBG‐control vector adenoviruses (Vector).

### mRNA‐seq Analyses

mRNA‐seq analyses on liver tissues were performed on mice (LIMA1 fl/fl and LIMA1 HKO; *n* = 5) fed with high‐fat‐diet for 20 weeks. Then the liver tissues and total RNA were collected for mRNA‐seq. Gene Denovo Biotechnology Co., Ltd, Guangzhou, performed all sample sequencing analyses.

### sEV^shCtr^ and sEV^shLIMA1^ Injection

Established HFD‐fed mice were randomly assigned into four groups. The groups included mice fed a normal chow diet with 10% kcal from fat (NCD group, *n* = 6), mice fed a high‐fat diet with 60% kcal from fat (HFD group, *n* = 6), HFD‐fed mice injected with sEV^shCtr^ (HFD/sEV^shCtr^ group, 2.4 × 10^9^ particles, *n* = 6), and HFD‐fed mice injected with sEV^shLIMA1^ (HFD/sEV^shLIMA1^ group, 2.4 × 10^9^ particles, *n* = 6). After 16 weeks, sEV^shCtr^ and sEV^shLIMA1^ were separately dissolved in saline solution and administered to the mice *i.v*. for 4 weeks. As a control, equivalent volumes of PBS were injected. All animals were euthanized after overnight fasting (12 h) at the end of the study. Blood samples collected from the retinal vein plexus were centrifuged (3500 rpm, 10 min, 4 °C) to separate the serum and then stored at ‐80 °C for further research.

### Statistical Analysis

All biological replicates were conducted with a minimum of three repetitions. The data presented show the mean value ± standard error of the mean. The normal distribution was determined using the Pearson normality test and D'Agostino test. Prism software (GraphPad 8.0, San Diego, CA) was used for statistical analysis. A one‐way ANOVA or Student's *t*‐test was utilized to assess significant differences between groups. Spearman Pearson correlation analysis was used to assess the association. All statistical tests were two‐tailed, and *p* < 0.05 was considered significant.

## Conflict of Interest

The authors declare no conflict of interest.

## Author Contributions

F.Y. and Y.C. contributed equally to this work. F.Y., Y.C., K.G., G.Z., L.F., T.L., and L.Z. is responsible for the Investigation and methodology. F.Y. is responsible for original draft writing. Y.Y. is responsible for conceptualization, data curation, funding acquisition, project administration, and supervision.

## Supporting information



Supporting Information

## Data Availability

The data that support the findings of this study are available from the corresponding author upon reasonable request.
