# Supplementary material for: LIMA1 O‐GlcNAcylation Promotes Hepatic Lipid Deposition through Inducing β‐catenin‐Regulated FASn Expression in Metabolic Dysfunction‐Associated Steatotic Liver Disease
Source: Adv Sci (Weinh). 2025 Feb 8;12(15):2415941. doi: 10.1002/advs.202415941 (PMC12005730; doi:10.1002/advs.202415941)
Supplement: Supplementary file 1 — Supporting Information [file ADVS-12-2415941-s001.docx]

Supporting Information

**LIMA1 O-GlcNAcylation Promotes Hepatic Lipid Deposition Through Inducing β-catenin-Regulated FASn Expression in Metabolic Dysfunction-Associated Steatotic Liver Disease**

*Fuji Yang*^1,2†^, *Yifei Chen*^1,2†^, *Guojun Zheng*^3^, *Kefeng Gu*^4^, *Lin Fan*^5^, *Tingfen Li*^6^, *Ling Zhu*^5^, *Yongmin Yan*^1,4,5*^

**This file includes:**

Figure S1 to S10

Table S1 to S2

**
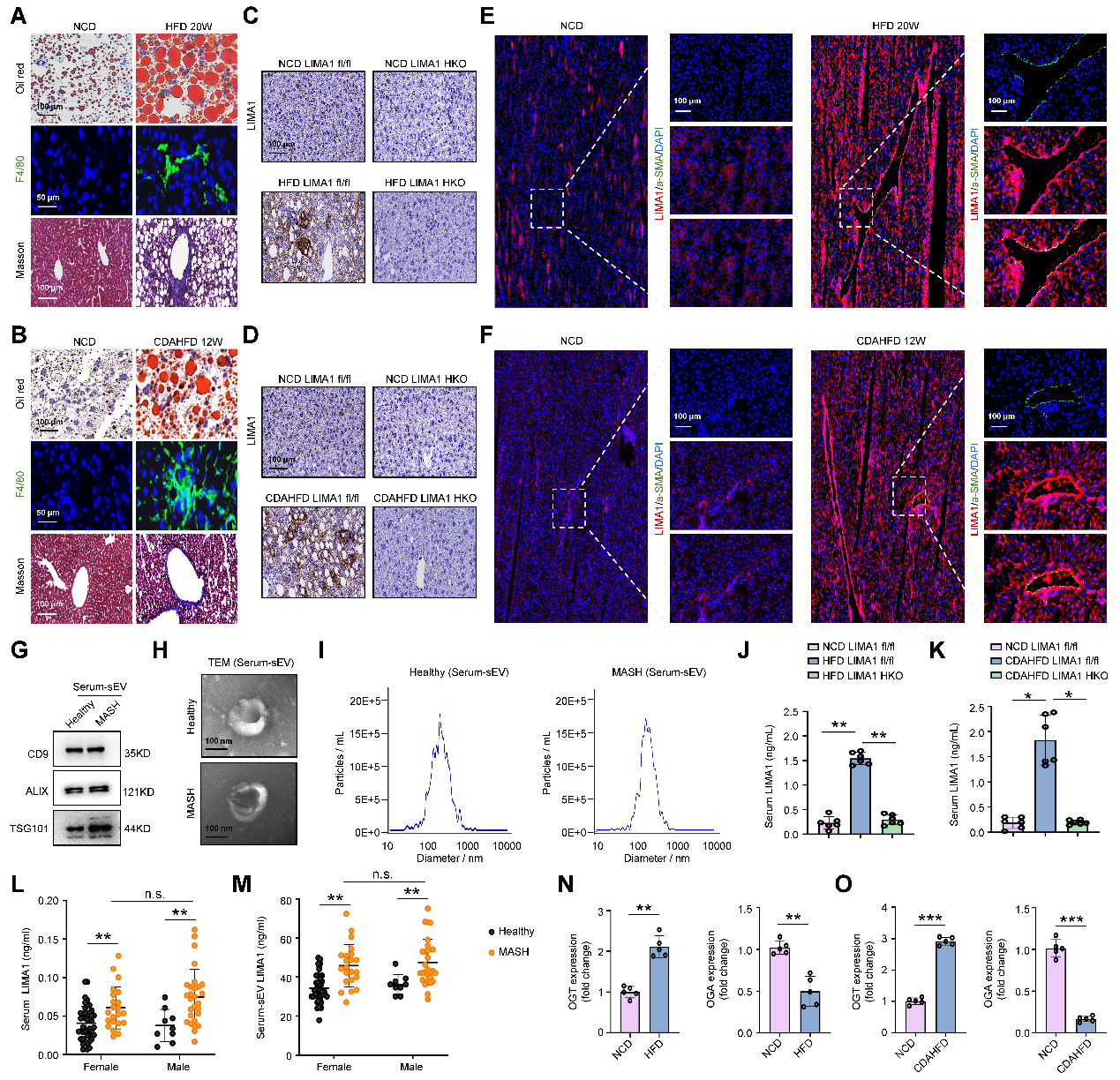
Supplementary Figure S1. Hepatocytes secreted LIMA1 and LIMA1 co-localization with activated HSC in liver of HFD-fed mice and CDAHFD-fed mice. A, B)** Oil red O (upper; Scale bars, 100 µm), F4/80 (middle; Scale bars, 50 µm), and Masson staining (bottom; Scale bars,100 µm) in livers from NCD-fed mice, HFD-fed mice, and CDAHFD-fed mice. **C, D)** Immuno-histochemistry of LIMA1 in livers from LIMA1 fl/fl and LIMA1 HKO mice fed with NCD, HFD, and CDAHFD. Scale bar, 100 µm. **E, F)** Immunofluorescence showing LIMA1 (red) and α-SMA (green) co-localization in livers from NCD-fed mice, HFD-fed mice, and CDAHFD-fed mice. Scale bar, 100 µm. **G)** Western blot analysis of CD9, Alix, and TSG101 in serum sEV isolated from healthy individuals and MASH patients. **H, I)** Representative transmission electron microscope (TEM) image and nanoparticle tracking analyses (NTA) result of serum sEV isolated from a healthy individual and MASH patients. Scale bar, 100 nm. **J, K)** Changes in serum LIMA1 levels from LIMA1 fl/fl and LIMA1 HKO mice fed with NCD, HFD, and CDAHFD (n = 6). **L, M)** A total of 50 healthy individuals and 50 MASH patients were categorized into gender groups. Changes in serum LIMA1 and serum-derived sEV LIMA1 levels in the indicated groups. **N, O)** Quantification of OGT and OGA protein expression in livers from NCD-fed mice, HFD-fed mice, and CDAHFD-fed mice (n = 5). The data were plotted as Mean ± SEM. * *p* < 0.05, ** *p* < 0.01, *** *p* < 0.001 by Student’s t-test for L, M, N and O; by one-way ANOVA for J and K.

**
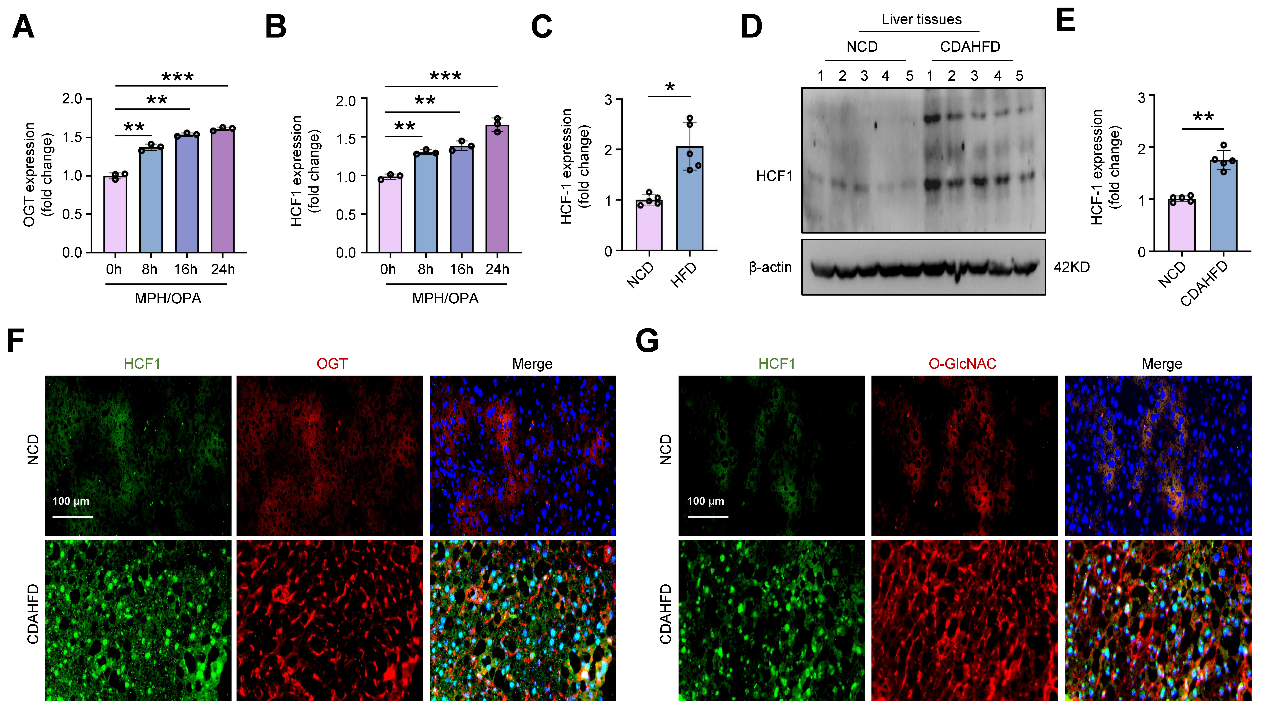
Supplementary Figure S2. OGT or HCF1 upregulation in OPA-treated MPH and HFD-fed mice and CDAHFD-fed mice.** **A, B)** Quantification of OGT and HCF1 protein expression in mouse primary hepatocytes (MPH) treated with OPA for 0, 8, 16, and 24 h (n = 3). **C)** Quantification of HCF1 protein expression in livers from NCD-fed mice and HFD-fed mice at 20 weeks (n = 5). **D, E)** Western blot analysis and quantification of HCF1 in livers from NCD-fed mice and CDAHFD-fed mice at 12 weeks (n = 5). **F, G)** Immunofluorescence showing HCF1 (green), OGT (red) or O-GlcNAc (red) colocalization in livers from NCD-fed mice and CDAHFD-fed mice. Scale bars, 100 µm. The data were plotted as Mean ± SEM. * *p* < 0.05, ** *p* < 0.01, *** *p* < 0.001 by Student’s t-test for C and E; by one-way ANOVA for A and B.

**
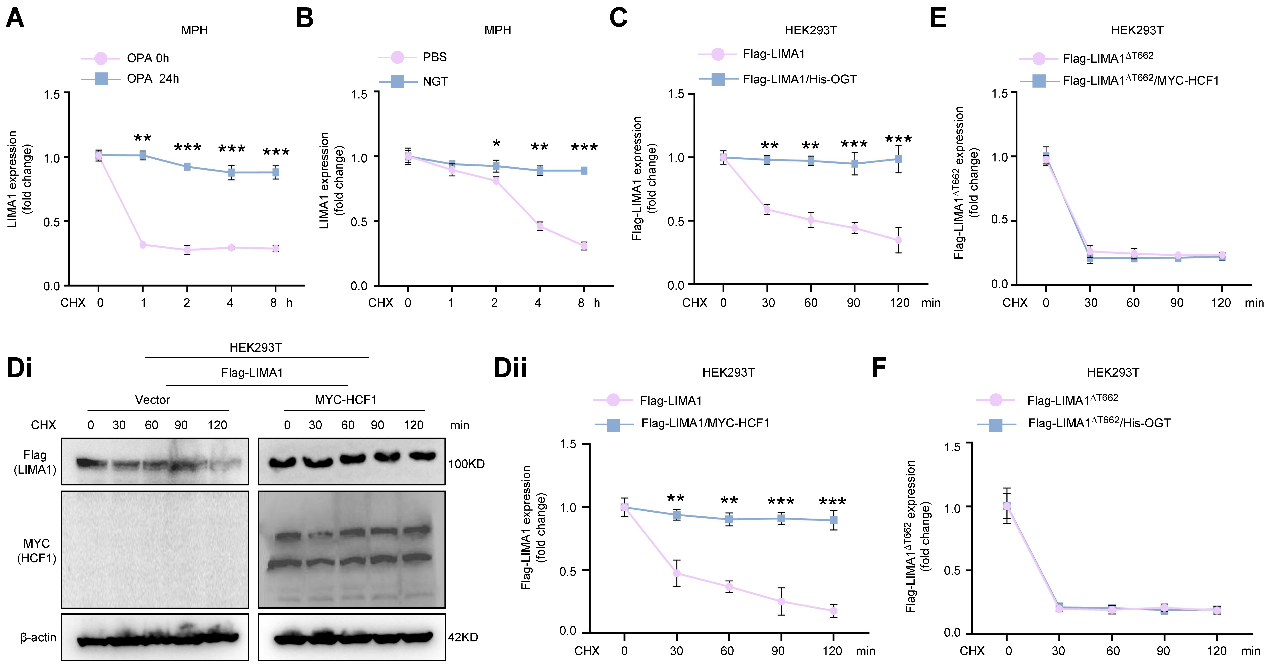
Supplementary Figure S3. HCF1/OGT complex-mediated O-GlcNAcylation of LIMA1 Thr662 enhances LIMA1 protein stability by counteracting its ubiquitylation. A, B)** Quantification of LIMA1 protein expression in MPH treated with PBS/OPA or PBS/NAG-thiazoline (NGT, 0.008 м) following cycloheximide (CHX, 20 µм) treatment for the indicated times, respectively (n = 3). **C)** Quantification of LIMA1 protein expression in HEK293T cells transfected with Flag-LIMA1/His-OGT following CHX (50 µм) treatment for the indicated times (n = 3). **D)** Cycloheximide chase assay showing that HCF1 overexpression increases LIMA1 stabilization. HEK293T cells were transfected with Flag-LIMA1/MYC-HCF1 and then exposed to CHX (50 μм) for the indicated times (n = 3). **E, F)** Quantification of LIMA1 protein expression in HEK293T cells transfected with Flag-LIMA1^∆T662^/MCF-HCF1 or Flag-LIMA1^∆T662^/His-OGT following CHX (50 µм) treatment for the indicated times. The data were plotted as Mean ± SEM. * *p* < 0.05, ** *p* < 0.01, *** *p* < 0.001 by Student’s t-test.

**
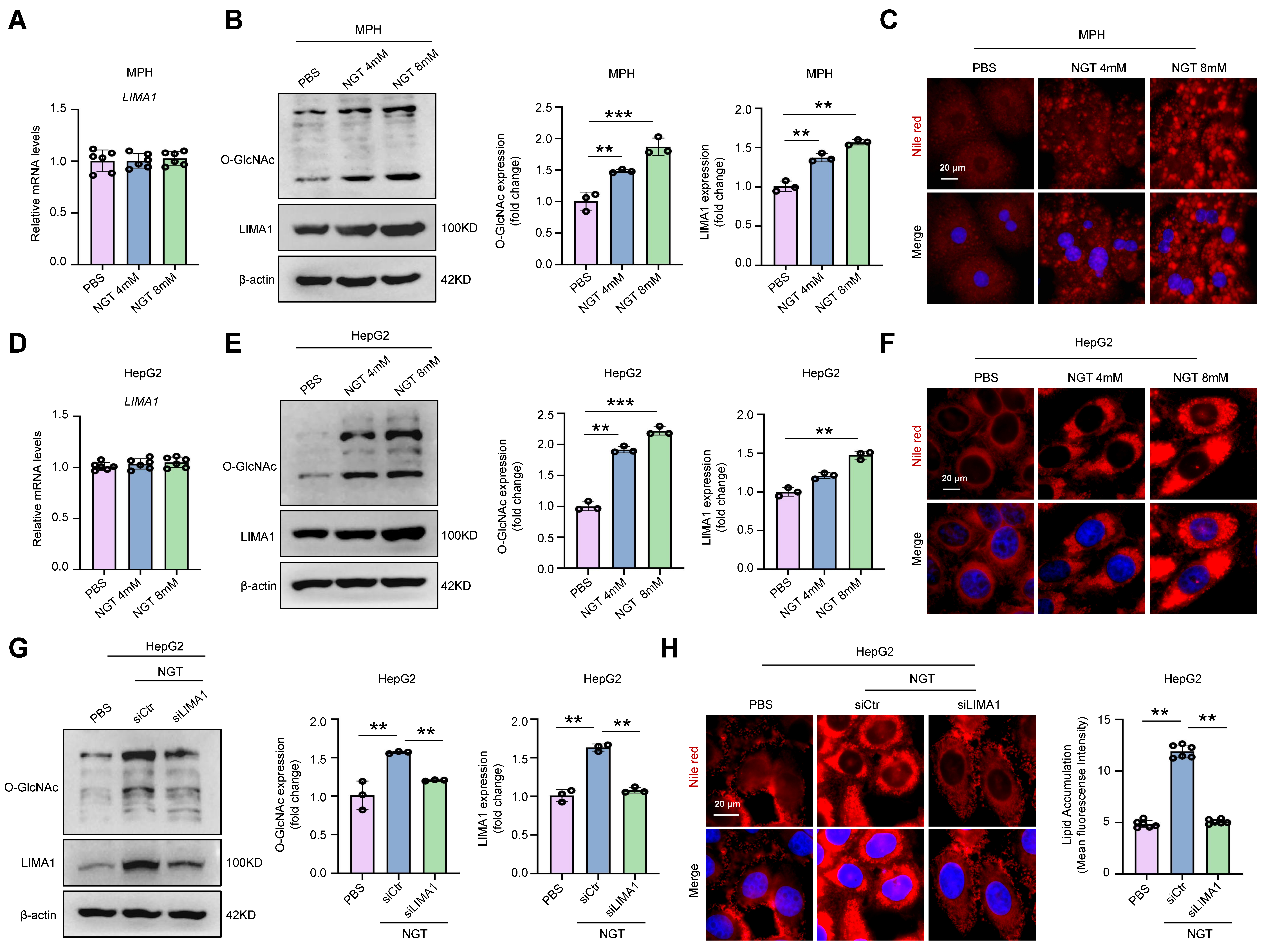
Supplementary Figure S4. O-GlcNAc increases LIMA1 expression and lipid deposition in hepatocytes. A)** Relative mRNA levels of LIMA1 in MPH treated with PBS or NGT (n = 6). **B)** Western blot analysis of O-GlcNAc and LIMA1 in MPH treated with PBS or NGT (n = 3). **C)** Nile red staining of intracellular lipid droplets in MPH treated with PBS or NGT. Scale bars, 20 µm. **D)** Relative mRNA levels of LIMA1 in HepG2 cells treated with PBS or NGT (n = 6). **E)** Western blot analysis of O-GlcNAc and LIMA1 in HepG2 cells treated with PBS or NGT (n = 3). **F)** Nile red staining of intracellular lipid droplets in HepG2 cells treated with PBS or NGT. Scale bars, 20 µm. **G)** Western blot analysis of O-GlcNAc and LIMA1 in normal HepG2 cells and NGT-treated HepG2 cells transfected with LIMA1 siRNA (siLIMA1) or control siRNA (siCtr) (n = 3). **H)** Nile red staining of intracellular lipid droplets in normal HepG2 cells and NGT-treated HepG2 cells transfected with siLIMA1 or siCtr (n = 6). Scale bars, 20 µm. The data were plotted as Mean ± SEM. * *p* < 0.05, ** *p* < 0.01, *** *p* < 0.001 by one-way ANOVA.

**
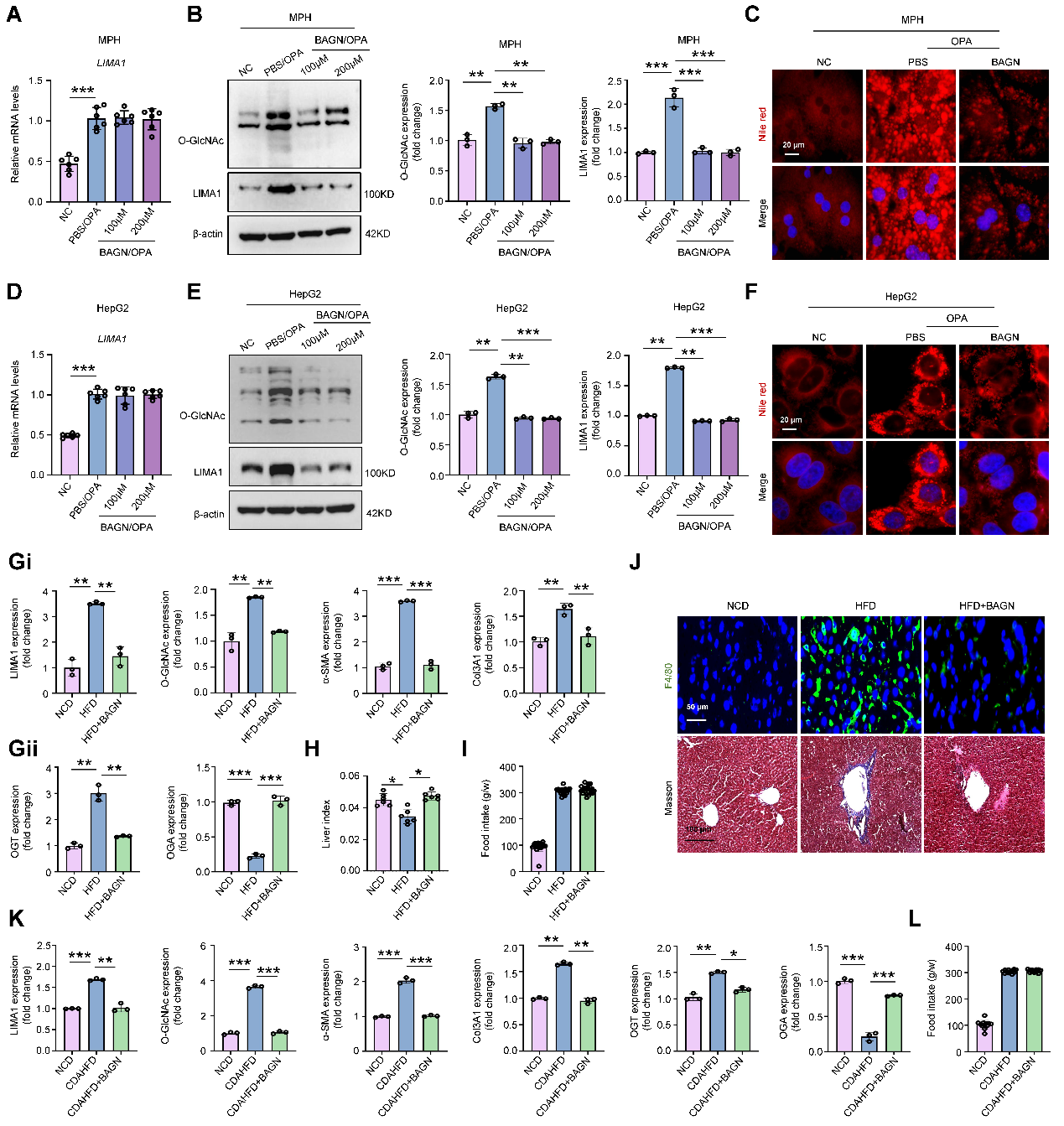
Supplementary Figure S5. Benzyl-α-GalNAc (BAGN) decreases LIMA1 expression and lipid deposition in hepatocytes. A)** Relative mRNA levels of LIMA1 in normal MPH and OPA-treated MPH treated with PBS or Benzyl-α-GalNAc (BAGN) (n = 6). **B)** Western blot analysis of O-GlcNAc and LIMA1 in normal MPH and OPA-treated MPH treated with PBS or BAGN (n = 3). **C)** Nile red staining of intracellular lipid droplets in normal MPH and OPA-treated MPH treated with PBS or BAGN. Scale bars, 20 µm. **D)** Relative mRNA levels of LIMA1 in normal HepG2 cells and OPA-treated HepG2 cells treated with PBS or BAGN (n = 6). **E)** Western blot analysis of O-GlcNAc and LIMA1 in normal HepG2 cells and OPA-treated HepG2 cells treated with PBS or BAGN (n = 3). **F)** Nile red staining of intracellular lipid droplets in normal HepG2 cells and OPA-treated HepG2 cells treated with PBS or BAGN. Scale bars, 20 µm. **G-I)** Quantification of relative protein expression in livers (n = 3) and changes in liver index (n = 6; liver index = liver wet weight/body weight), food intake (n = 20) from NCD-fed mice, HFD-fed mice, and HFD-fed mice injected with BAGN. **J)** F4/80 (Scale bars, 50 µm) and Masson (Scale bars, 100 µm) staining in livers from NCD-fed mice, HFD-fed mice, and HFD-fed mice injected with BAGN. **K, L)** Quantification of relative protein expression in livers (n = 3) and changes in food intake (n = 12) from NCD-fed mice, CDAHFD-fed mice, and CDAHFD-fed mice injected with BAGN. The data were plotted as Mean ± SEM. * *p* < 0.05, ** *p* < 0.01, *** *p* < 0.001 by one-way ANOVA.

**
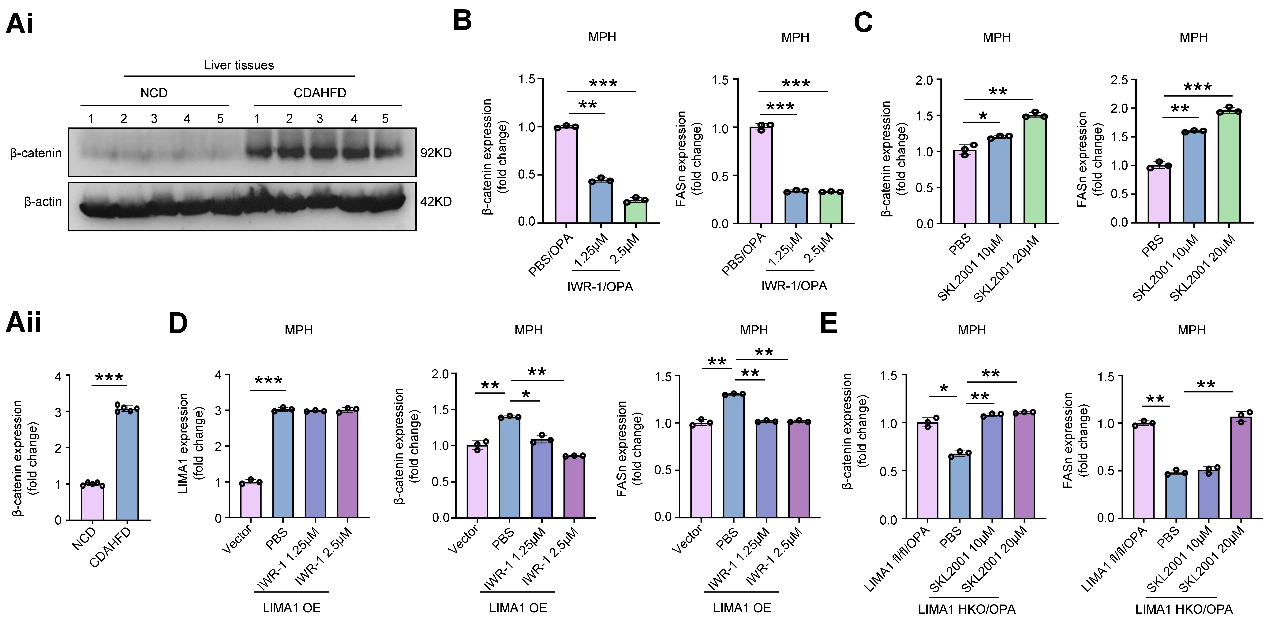
Supplementary Figure S6. LIMA1 promotes FASn-mediated lipid deposition in hepatocytes by β-catenin. A)** Western blot analysis of β-catenin in livers from NCD-fed mice and CDAHFD-fed mice at 12 weeks (n = 5). **B, C)** Quantification of β-catenin and FASn protein expression in PBS or IWR-1 treated OPA-injured MPH, and PBS or SKL2001 treated MPH (n = 3). **D)** Quantification of LIMA1, β-catenin, and FASn protein expression in control vector transfected MPH (vector) and pADV-CMV-LIMA1-Flag transfected MPH (LIMA1 OE) treated with or without IWR-1 for 24 h (n = 3). **E)** Quantification of β-catenin and FASn protein expression in OPA-treated LIMA1 fl/fl MPH and OPA-treated LIMA1 HKO MPH treated with or without SKL2001 for 24 h (n = 3). The data were plotted as Mean ± SEM. * *p* < 0.05, ** *p* < 0.01, *** *p* < 0.001 by Student’s t-test for A; by one-way ANOVA for B, C, D and E.

**
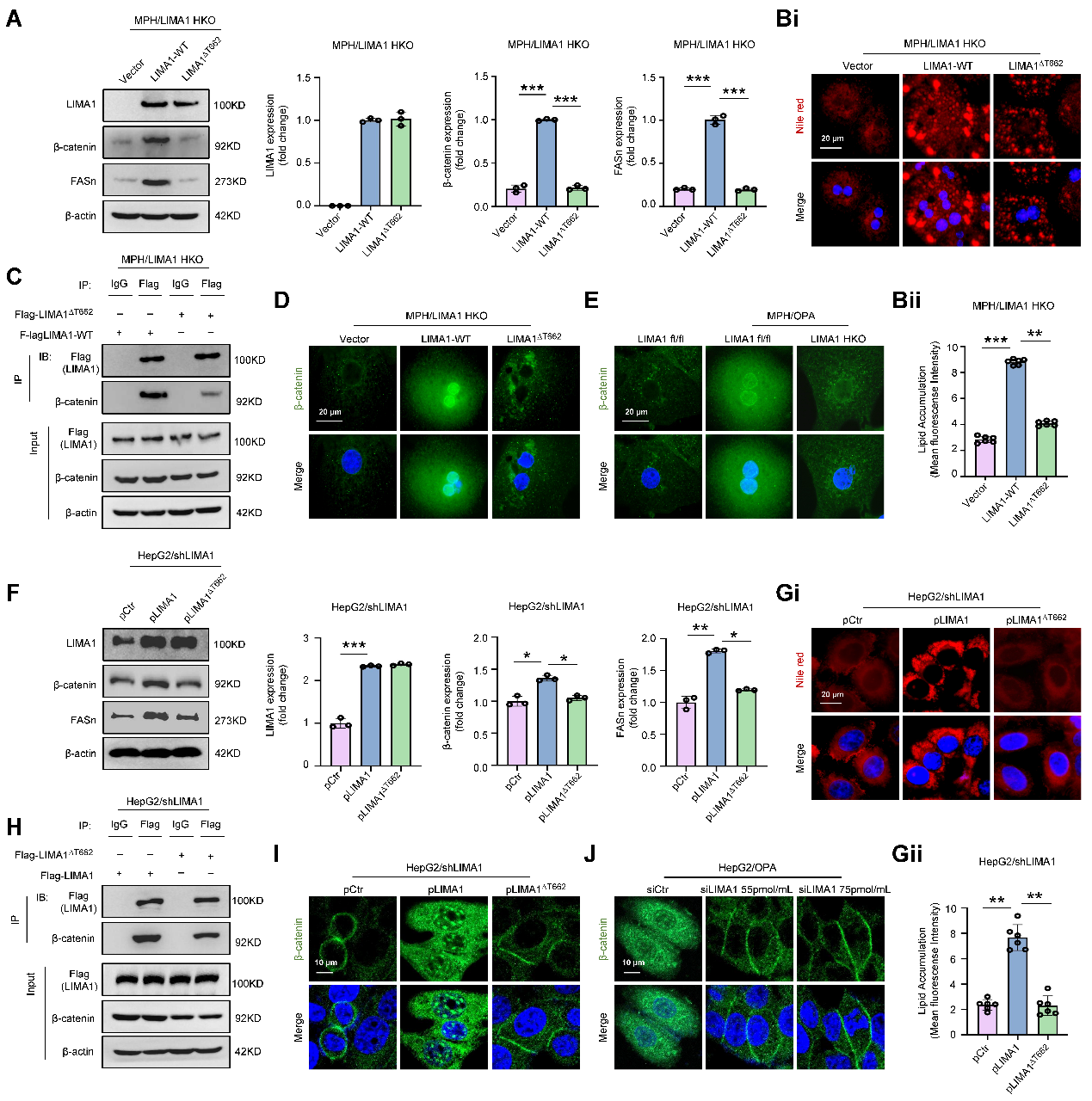
Supplementary Figure S7. T662 mutant inactivates LIMA1 induced β-catenin/FASn signaling and lipid deposition in hepatocytes. A)** At 7 weeks of age, male LIMA1 HKO mice were injected in the tail vein with adeno-associated virus type 8 (AAV8)-TBG-LIMA1-WT-Flag vector (LIMA1-WT) or AAV8-TBG-LIMA1-T662A-Flag vector (LIMA1^∆T662^), and LIMA1 HKO mice injected with AAV8-TBG-control vector were used as controls. MPH isolated from the indicated mouse groups. Western blot analysis of LIMA1, β-catenin, and FASn in LIMA1 HKO MPH with control vector or LIMA1-WT-Flag or LIMA1^∆T662^-Flag overexpression (n = 3). **B)** Nile red staining of intracellular lipid deposition in each group (n = 6). Scale bars, 20 µm. **C)** Interaction between LIMA1 and β-catenin by Co-IP in LIMA1 HKO MPH with LIMA1-WT-Flag or LIMA1^∆T662^-Flag overexpression. After immunoprecipitation with Flag antibodies, immunoprecipitates were analyzed using an anti-β-catenin antibody. **D)** Immunofluorescence of β-catenin (green) in LIMA1 HKO MPH from the indicated groups. Scale bars, 20 µm. **E)** Immunofluorescence of β-catenin (green) in LIMA1 fl/fl MPH, OPA-treated LIMA1 fl/fl MPH, and OPA-treated LIMA1 HKO MPH. Scale bars, 20 µm. **F)** Western blot analysis of LIMA1, β-catenin, and FASn in HepG2^shLIMA1^ cells transfected with pCtr or pLIMA1 or pLIMA1^∆T662^ (n = 3). **G)** Nile red staining of intracellular lipid deposition in HepG2^shLIMA1^ cells transfected with pCtr or pLIMA1 or pLIMA1^∆T662^ (n = 6). Scale bars, 20 µm. **H)** Interaction between LIMA1 and β-catenin by Co-IP in HepG2^shLIMA1^ cells transfected with Flag-LIMA1 and Flag-LIMA1^ΔT662^. After immunoprecipitation with Flag antibodies, immunoprecipitates were analyzed using an anti-β-catenin antibody. **I)** Immunofluorescence of β-catenin (green) in HepG2^shLIMA1^ cells transfected with pCtr or pLIMA1 or pLIMA1^∆T662^. Scale bars, 10 µm. **J)** Immunofluorescence of β-catenin (green) in OPA-treated HepG2 cells transfected with siCtr or siLIMA1. Scale bars, 10 µm. The data were plotted as Mean ± SEM. * *p* < 0.05, ** *p* < 0.01, *** *p* < 0.001 by one-way ANOVA.

**
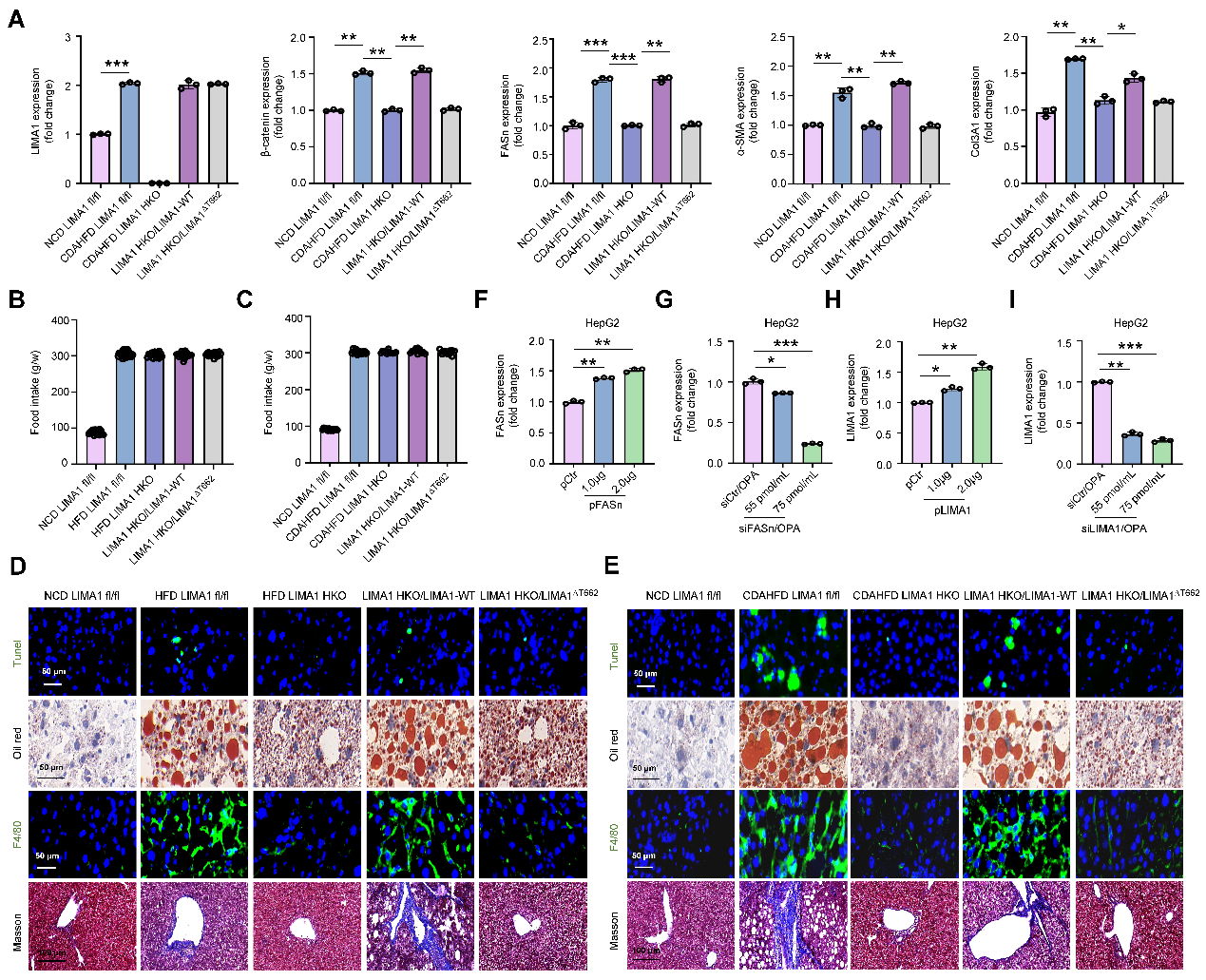
Supplementary Figure S8. Inhibiting LIMA1 O-GlcNAcylation inactivates LIMA1 induced β-catenin/FASn signaling in diet-induced MASLD. A)** Quantification of LIMA1, β-catenin, FASn, α-SMA, and Col3A1 in livers from NCD-fed LIMA1 fl/fl mice, CDAHFD-fed LIMA1 fl/fl mice, CDAHFD-fed LIMA1 HKO mice, CDAHFD-fed LIMA1 HKO mice injected AAV8-TBG-LIMA1-WT-Flag vector or AAV8-TBG-LIMA1-T662A-Flag vector (n = 3). **B, C)** Changes in food intake from mice in the indicated groups. **D-E)** Tunel (Scale bars, 50 µm), Oil red O (Scale bars, 50 µm), F4/80 (Scale bars, 50 µm), and Masson (Scale bars, 100 µm) staining in livers from the indicated mouse groups. **F)** Quantification of FASn protein expression in HepG2 cells transfected with pCtr or pFASn (n = 3). **G)** Quantification of FASn protein expression in OPA-treated HepG2 cells transfected with siCtr or siFASn (n = 3). **H)** Quantification of LIMA1 protein expression in HepG2 cells transfected with pCtr or pLIMA1 (n = 3). **I)** Quantification of LIMA1 protein expression in OPA-treated HepG2 cells transfected with siCtr or siLIMA1 (n = 3). The data were plotted as Mean ± SEM. * *p* < 0.05, ** *p* < 0.01, *** *p* < 0.001 by one-way ANOVA.

**
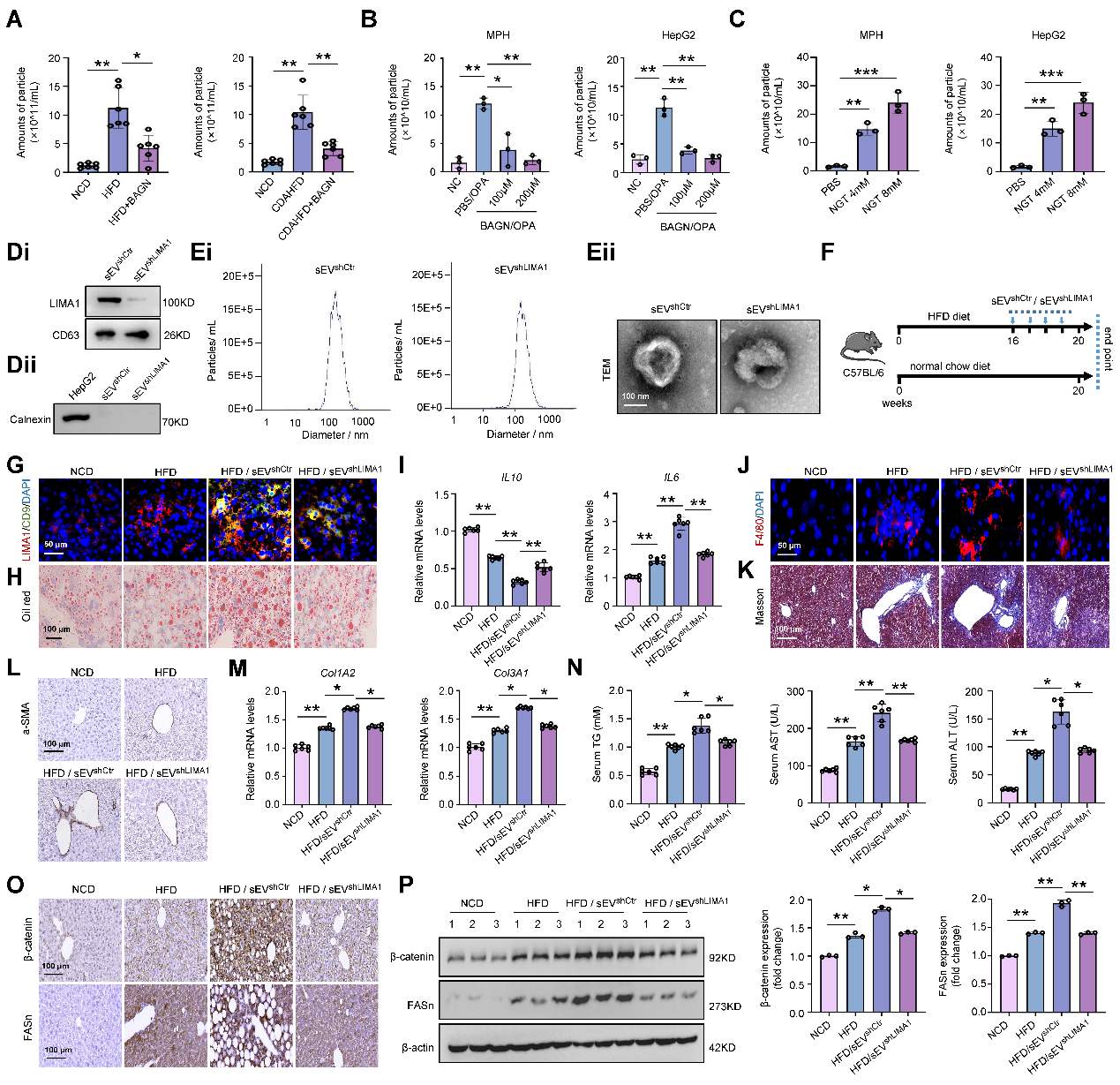
Supplementary Figure S9. LIMA1 knockdown reduces LTH-sEV promoted MASLD progression in HFD-fed mice. A)** Changes in serum sEV levels of NCD-fed mice, HFD-fed mice, and HFD-fed mice injected with BAGN in each group (n = 6). **B)** Changes in sEV levels in normal MPH/HepG2 cells and OPA-injured MPH/HepG2 cells treated with PBS or BAGN (n = 3). **C)** Changes in sEV levels in MPH/HepG2 cells treated with PBS or NGT (n = 3). **D)** LIMA1, CD63, or Calnexin expression in steatotic HepG2 cells, LTH-sEV^shCtr^ from steatotic HepG2^shCtr^ cells (instead by sEV^shCtr^), and LTH-sEV^shLIMA1^ (instead by sEV^shLIMA1^) from steatotic HepG2^shLIMA1^ cells was examined by western blotting. **E)** Representative result of nanoparticle tracking analyses (NTA) and transmission electron microscope (TEM) of sEV^shCtr^ and sEV^shLIMA1^. scale bar, 100 nm. **F)** C57BL/6 mice were placed on high-fat diet (HFD, 60%) or normal chow diet and injected with sEV^shCtr^ (2.4×10^9^ particles) or sEV^shLIMA1^ (2.4×10^9^ particles) from the 16th week to the 20th week of HFD feeding. As a control, the same volume of PBS was injected. **G)** Immunofluorescence showing LIMA1 (red) and CD9 (green) colocalization in livers from NCD-fed mice, HFD-fed mice, and HFD-fed mice injected with sEV^shCtr^ or sEV^shLIMA1^. Scale bars, 50 µm. **H)** Oil red O staining in livers from from NCD-fed mice, HFD-fed mice, and HFD-fed mice injected with sEV^shCtr^ or sEV^shLIMA1^. Scale bars, 100 µm. **I)** Relative mRNA levels of IL-10 and IL-6 in livers from NCD-fed mice, HFD-fed mice, and HFD-fed mice injected with sEV^shCtr^ or sEV^shLIMA1^ (n = 6). **J)** Immunofluorescence of F4/80 (red) in livers from NCD-fed mice, HFD-fed mice, and HFD-fed mice injected with sEV^shCtr^ or sEV^shLIMA1^. Scale bars, 50 µm. **K)** Masson staining in livers from NCD-fed mice, HFD-fed mice, and HFD-fed mice injected with sEV^shCtr^ or sEV^shLIMA1^. Scale bars, 100 µm. **L)** Immunohistochemistry of α-SMA in livers from NCD-fed mice, HFD-fed mice, and HFD-fed mice injected with sEV^shCtr^ or sEV^shLIMA1^. Scale bars, 100 µm. **M)** Relative mRNA levels of Col1A2 and Col3A1 in livers from NCD-fed mice, HFD-fed mice, and HFD-fed mice injected with sEV^shCtr^ or sEV^shLIMA1^ (n = 6). **N)** Serum triglycerides (TG), aspartate transaminase (AST), and alanine aminotransferase (ALT) levels in NCD-fed mice, HFD-fed mice, and HFD-fed mice injected with sEV^shCtr^ or sEV^shLIMA1^ (n = 6). **O-P)** Immunohistochemistry and Western blot analysis of β-catenin and FASn in livers from NCD-fed mice, HFD-fed mice, and HFD-fed mice injected with sEV^shCtr^ or sEV^shLIMA1^ (n = 3). Scale bars, 100 µm. The data were plotted as Mean ± SEM. * *p* < 0.05, ** *p* < 0.01, *** *p* < 0.001 by one-way ANOVA.

**
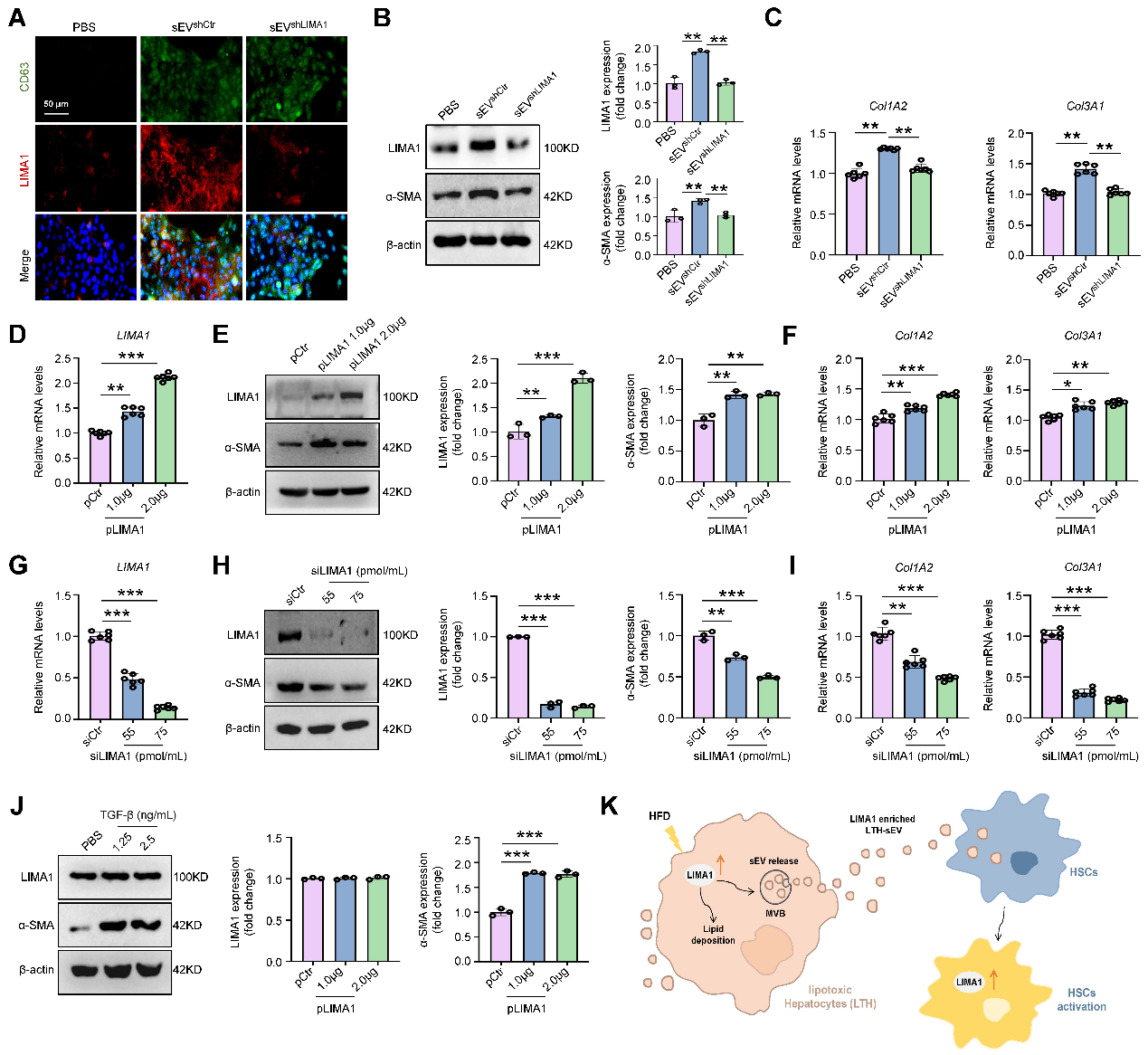
Supplementary Figure S10. LIMA1 knockdown reduces LTH-sEV promoted HSC activation in vitro.** **A)** Immunofluorescence showing CD63 (green) and LIMA1 (red) co-localization in LX2 cells treated with sEV^shCtr^ (1.2×10^8^ particles), sEV^shLIMA1^ (1.2×10^8^ particles) or PBS for 24 h. Scale bars, 50 µm. **B)** Western blot analysis of LIMA1 and α-SMA in LX2 cells treated with sEV^shCtr^, sEV^shLIMA1^ or PBS (n = 3). **C)** Relative mRNA levels of Col1A2 and Col3A1 in LX2 cells treated with sEV^shCtr^, sEV^shLIMA1^ or PBS (n = 6). **D-F)** Relative mRNA levels (n = 6) and Western blot analysis (n = 3) of LIMA1 or α-SMA or Col1A2 and Col3A1 in LX2 cells transfected with pCtr or pLIMA1. **G-I)** Relative mRNA levels (n=6) and Western blot analysis (n = 3) of LIMA1 or α-SMA or Col1A2 and Col3A1 in LX2 cells transfected with siCtr or siLIMA1. **J)** Western blot analysis of LIMA1 and α-SMA in LX2 cells treated with TGF-β or PBS (n = 3). **K)** Schematic representation of LTH-sEV-delivered LIMA1 enhanced MASLD progression by promoting hepatic stellate cell (HSC) activation. The data were plotted as Mean ± SEM. * *p* < 0.05, ** *p* < 0.01, *** *p* < 0.001 by one-way ANOVA.

| **Table S1. Base characteristics of patients with biopsy-proven MASH** | | | |  |
| --- | --- | --- | --- | --- |
|  |  |  |  |  |
| Characteristics | Total  (n=50) | Gender | |  |
|  |  | Male (n=29) | Female (n=21) |  |
| Age [year] | 36 (10, 70) | 36 (10, 53) | 37 (20, 70) |  |
| BMI [kg m^2 -1^] | 28 (20.8, 38) | 28.55 (23.7, 38) | 26.3 (20.8, 29.3) |  |
| ALT [U L^-1^] | 58.75 (15.9, 424.6) | 64.1 (18.5, 424.6) | 52.7 (15.9, 220) |  |
| AST [U L^-1^] | 40 (12, 252) | 39 (17, 252) | 41 (12, 126) |  |
| GGT [U L^-1^] | 64.6 (16.2, 844) | 64 (16.2, 337.6) | 65.2 (18.1, 258.1) |  |
| ALP [U L^-1^] | 77 (48, 171) | 79 (52, 171) | 76.5 (48, 122) |  |
| TG [mmol L^-1^] | 1.72 (0.57, 11.5) | 1.74 (0.87, 11.5) | 1.7 (0.57, 6.25) |  |
| TC [mmol L^-1^] | 4.985 (1.34, 8.08) | 4.98 (1.63, 8.08) | 4.985 (1.34, 6.54) |  |
| FG [mmol L^-1^] | 5.3 (3.92, 15.37) | 5.265 (3.92, 15.37) | 5.23 (4.21, 7.8) |  |
| Platelet [×10^9^ L^-1^] | 230 (146, 439) | 232 (146, 439) | 232 (167, 436) |  |
| CAP [db m^-1^] | 331.5 (223, 380) | 342 (286, 380) | 319 (223, 360) |  |
| FibroScan [kPa] | 6.65 (4.1, 21.5) | 6.65 (4.4, 12.4) | 6.65 (4.1, 21.5) |  |
| Abbreviations: ALT, alanine transaminase; AST, aspartate transaminase; ALP, alkaline phosphatase; BMI, body mass index; CAP, controlled attenuation parameter; FG, fast glycemia; GGT, γ-glutamyltransferase; MASH, metabolic dysfunction-associated steatohepatitis; NAS, nonalcoholic fatty liver disease activity score; TG, triglycerides; TC, total cholesterol. | | | |  |
|  |  |  |  |  |
|  |  |  |  |  |
|  |  |  |  |  |
|  |  |  |  |  |

**Table S2. Primers for qPCR detection**

| Gene (Mus) |  | Sequence 5'---3' |
| --- | --- | --- |
| *β-actin* | F | GTGACGTTGACATCCGTAAAGA |
|  | R | GCCGGACTCATCGTACTCC |
| *IL-10* | F | CCAAGCCTTATCGGAAATGA |
|  | R | TTTTCACAGGGGAGAAATCG |
| *IL-6* | F | AGTTGCCTTCTTGGGACTGA |
|  | R | TCCACGATTTCCCAGAGAAC |
| *Col1A2* | F | TGAGACAGGCGAACAAGGTG |
|  | R | GCTGAGGCAGGAAGCTGAAG |
| *Col3A1* | F | CTGGTCAGCCTGGAGATAAG |
|  | R | ACCAGGACTACCACGTTCAC |
| *FABP1* | F | TGGTCCGCAATGAGTTCACCCT |
|  | R | CCAGCTTGACGACTGCCTTGACTT |
| *CD36* | F | TGGGTTTTGCACATCAAAGA |
|  | R | GATGGACCTGCAAATGTCAGA |
| *PPAR-α* | F | TATTCGGCTGAAGCTGGTGTAC |
|  | R | CTGGCATTTGTTCCGGTTCT |
| *CPT-1A* | F | AGGACCCTGAGGCATCTATT |
|  | R | ATGACCTCCTGGCATTCTCC |
| *FASn* | F | GCTGTCCTGCCTCTGGTGCTTGCTG |
|  | R | AGGTTGGTGTACCCCCATTCATTTT |
| *SCD1* | F | CGCTGGCACATCAACTTCAC |
|  | R | AGGAACTCAGAAGCCCAAAGC |
| *LIMA1* | F | TCTAGTCAGCCCACAGGTGTCT |
|  | R | CTCTTCTAAAGTGCTGGGGCA |
| Gene (Homo) |  | Sequence 5'---3' |
| *β-actin* | F | TCCTTCCTGGGCATGGAGT |
|  | R | CAGGAGGAGCAATGATCTTGAT |
| *LIMA1* | F | AAGTACCAGGCAGCTGTGTC |
|  | R | AGTCACCTGGGGAGTCATCT |
| *Col1A2* | F | AGCAGGAGGTTTCGGCTAAG |
|  | R | GCA ACA AAGTCCGCGTATCC |
| *Col3A1* | F | CGCCCTCCTAATGGTCAAGG |
|  | R | TTCTGAGGACCAGTAGGGCA |
